# Additive effects of emotional expression and stimulus size on the perception of genuine and artificial facial expressions: an ERP study

**DOI:** 10.1038/s41598-024-55678-2

**Published:** 2024-03-06

**Authors:** Annika Ziereis, Anne Schacht

**Affiliations:** Department for Cognition, Emotion and Behavior, Affective Neuroscience and Psychophysiology Laboratory, Georg-August-University of Göttingen, 37073 Göttingen, Germany

**Keywords:** Psychology, Emotion

## Abstract

Seeing an angry individual in close physical proximity can not only result in a larger retinal representation of that individual and an enhanced resolution of emotional cues, but may also increase motivation for rapid visual processing and action preparation. The present study investigated the effects of stimulus size and emotional expression on the perception of happy, angry, non-expressive, and scrambled faces. We analyzed event-related potentials (ERPs) and behavioral responses of *N* = 40 participants who performed a naturalness classification task on real and artificially created facial expressions. While the emotion-related effects on accuracy for recognizing authentic expressions were modulated by stimulus size, ERPs showed only additive effects of stimulus size and emotional expression, with no significant interaction with size. This contrasts with previous research on emotional scenes and words. Effects of size were present in all included ERPs, whereas emotional expressions affected the N170, EPN, and LPC, irrespective of size. These results imply that the decoding of emotional valence in faces can occur even for small stimuli. Supra-additive effects in faces may necessitate larger size ranges or dynamic stimuli that increase arousal.

## Introduction

Encountering an individual staring at you with anger requires rapid recognition, appraisal, and response—particularly if the person is in close proximity. Over decades, research has accumulated evidence supporting preferential processing of emotional facial expressions compared to neutral expressions^1–4^, likely attributable to their biological relevance^5^. However, several boundary conditions have been identified to moderate emotion effects, such as task demands^4,6,7^ or reappraisal^8,9^. As face perception is strongly context-dependent^10^, (perceived) physical distance and an accurate representation of emotional cues modulates the processing of faces and their relevance. Thus, the main goal of the present study was to systematically investigate how the perception of emotional facial expressions is influenced by stimulus size. We presented faces showing real or manipulated happy, angry, or neutral expressions of different sizes and implemented a go/no-go task, in which participants judged the naturalness of the expression. Additionally, we incorporated scrambled faces in different sizes as emotionally meaningless control stimuli (no-go condition).

There are at least two different ways stimulus size may affect the neurophysiological processing of faces: First, especially early visual processing is influenced by several stimulus features like luminance^11^, contrast^12^, and spatial frequencies^13^, which can also be indirectly influenced by the retinal size of a stimulus^14^. Hence, the visual processing of the same arbitrary object varies when presented at different distances, attributed to changes in its retinal size. Second, stimulus size correlates with perceived physical proximity and distance, at least for stimuli like faces whose real size is known^15^. Previous research suggests that the perception of biologically relevant stimuli, such as faces, is enhanced as stimulus size increases. For example, pictures of emotional and neutral scenes produced differences in autonomic responses depending on the size they were presented in^16^. Similarly, skin conductance effects were greater between arousing and non-arousing video clips presented on large screens compared to small screens^17^. Furthermore, the physical distance of faces expressing emotions has been shown to affect physiological activations, such as pupil diameter^18^, facial mimicry^18^, and heart rate (variability)^19^. The size of faces has been shown to modulate emotion judgements^20^^, cf.^^21^ and eye movements^22^. Even memory formation appears to be influenced by image size, suggesting a contribution of low- and high-level visual processes^23^. Increased emotion effects as a function of increased stimulus size may partly be attributed to greater perceptual salience and indirectly to enhanced processing of relevant features, such as detecting nuanced emotional cues in facial expressions.

Notably, few studies have used event-related potentials (ERPs) to examine the influence of stimulus size on the neurophysiological processing of emotional stimuli (e.g., words^24^; emotional scenes^25^; feedback processing^26^; looming faces^27,28^; faces with administered pain^29^; and peripherally threatening fearful and neutral faces^30^) over time. Two studies reported interactions between stimulus size and emotional valence at mid-latency occipito-temporal components: for pleasant compared to neutral scenes between 150 and 300 ms^25^; for positive and negative compared to neutral words between 340 and 480 ms^24^, whereas no interactions at early and later processing stages were found. In contrast, enhanced early effects (P1) have been reported for looming (i.e., size-increasing) angry compared with neutral faces^27,28^, and also the face-sensitive N170 has shown to be elevated for looming fearful compared with neutral faces^30^. Moreover, later effects (P3) were reported to be modulated by size for pain-administered faces^29^. These findings suggest that both early and late processing of emotional information in faces might be affected by size. Early visual components of face processing are generally known to be sensitive to size^31–33^. However, the evidence regarding a sensitivity to emotional expressions is inconclusive^4^, leaving open the question whether supra-additive effects of emotion and stimulus size exist beyond mere detectability, i.e., when a face reaches a certain size threshold to be recognized as a face. Moreover, despite the large body of research on emotional face perception, to our knowledge, a systematic investigation of potential interactions between emotional expressions and presentation sizes of faces on early to late processing is still lacking.

With the present study, we aimed to contribute to a better understanding of the emotion specificity of commonly reported ERPs in face perception and to identify a possible reason for the heterogeneous findings of early emotion effects in the literature, particularly regarding the P1^4^: The P1 is an occipital positivity with a bilateral distribution and typically peaking around 100 ms after the onset of a visual stimulus. Only a minority of studies have reported modulations of the P1 by facial expressions^1,4,34–36^, whereas its sensitivity to size has been reported in studies of the perception of other, more abstract stimuli^37,38^. The N170, a negative deflection over occipito-temporal regions peaking at around 170 ms, has been related to face perception due to its enhancement for faces compared to other visual objects^39,40^, and has been shown to be size-sensitive^32^. Emotion-related effects on the EPN, the early posterior negativity, have been reported not only for faces but also for other stimulus domains^41,42^, which is why it is generally assumed to reflect selective attention to hedonic and arousing stimuli^43^. While the EPN has been suggested to reflect rather automatic processing, emotion effects on the late positive complex/potential (LPC/LPP) have been reported more robustly for tasks requiring attention to the affective content of stimuli^4^ and are known to be sensitive to task demands^6,44^. To encourage the processing of facial expressions without explicitly deciding on their emotional valence, in the present study, we implemented a task in which participants had to judge the naturalness of facial expressions.

Overall, we hypothesized that different stages of face processing would be differentially affected by stimulus size, with stronger size effects independent of emotional content in early processing, and size-emotion interactions appearing more during later processing stages of relevance appraisal. Additionally, we hypothesized that the influence of size would depend on whether its internal representation is beneficial for the current task goals, due to the general task dependency of later processing. Moreover, based on the biological relevance hypothesis^5^, all size × emotion interactions should consist of greater differences between emotional and neutral expressions for larger faces compared to smaller faces.

Specifically, we expected main effects of size on the P1, with larger peak amplitudes and shorter latencies for larger stimuli (similar to^38^) and differences between scrambled and intact faces due to the differences in contrast configuration. We were particularly interested in whether emotion effects on the P1 would emerge only in large faces, but not or only to a lesser extent, in small faces, thus partially explaining the inconsistent findings of emotion effects in the P1 research^4^. For the N170, we expected to replicate the face vs. non-face effect^39^ between intact and scrambled stimuli, which we also tested for differential modulation by size. As latency effects might be carried over from earlier processing, we expected a latency effect of size on N170 peak amplitudes. Furthermore, faces with emotional expressions, especially angry faces, should produce a more pronounced N170 compared to faces with neutral expressions^4^. Based on related research^24,25^ we predicted a main effect of size with more negative amplitudes for larger stimuli, a main effect of emotion with more negative amplitudes for happy and angry faces compared to neutral faces, and an interaction between emotion and size with stronger emotion effects for larger stimuli for the EPN. In addition, we predicted that intact faces would elicit larger amplitudes compared to scrambled stimuli due to their higher (task) relevance. The amplitude of the LPC component is expected to be modulated by size, with larger stimuli resulting in larger positive amplitudes. We expected that the naturalness decision on large faces would be made with higher and sustained motivation than on small faces because facial features would be presented in more detail. We also expected a main effect of emotion, with larger amplitudes for happy and angry compared to neutral expressions, because our task required focusing on and judging the facial expressions, although the specific emotional valence was not task-relevant.

We did not have a directed hypothesis regarding the impact of stimulus size on response times (RT), since previous research has been inconclusive^25,37^. Similarly, the effects of emotion on RTs seem to depend on the specific task demands. Several studies reported processing advantages for angry faces, reflected in faster responses^45,46^, whereas delayed disengagement from angry faces might lead to slower responses^42^. With respect to emotion, the naturalness decision for neutral faces should be rather difficult, as the original stimuli were only subtly altered. Thus, we expected that happy faces would be the fastest to respond to and show the highest accuracy, without specifying differences between neutral and angry faces. Furthermore, we did not have directed hypotheses regarding the effect of the manipulation (real vs. fake expressions) on the behavioral measures and ERPs. All cases in which interaction effects with the other variables have been incorporated into the results section. The full exploratory analyses can be found in the Supplementary Information. Due to the nature of the task, we did not expect a high rate of false alarms to scrambled images and therefore excluded the no-go trials from the analysis of the behavioral data.

## Method

We preregistered this study on https://osf.io/evfks.

### Participants

Data were analyzed from 40 participants (29 female, 11 male, 0 diverse; *M*_age_ = 22.98 years, *SD*_age_ = 3.23), our pre-registered sample size. Of the 43 participants originally enrolled, three data sets had to be excluded due to excessive artifacts, resulting in fewer than 30 trials per condition. All participants had good German language skills, were right-handed (according to self-assessment^47^), and reported no (neuro-) psychiatric disorders. We only included participants with normal or within plus/minus one diopter corrected-to-normal vision. Participants were recruited through the department’s participant recruitment database, postings on social media (Twitter, Facebook), and the university’s and institute’s online bulletin board. Participants were compensated at an hourly rate or an equivalent amount of course credits.

### Stimuli

The faces were selected from the Göttingen face database (GFD)^48^ and the Radboud face database^49^, cropped and combined with a transparency mask covering the hairline, ears, and neck. Of the 90 face identities, 45 showed the original expression (neutral, angry and happy, 15 each), while the other 45 (all of which had a neutral expression in their original version) were manipulated using a generative adversarial network (GAN) to create instances of neutral, angry, and happy expressions (15 each) by scaling the intensities of selected action units (see section “Creating fake expressions”). Stimuli were presented in grayscale on a light gray background in three different sizes at a viewing distance of approximately 78 cm. Measured sizes were 8.3 $\times$ 5.8 cm (6.09 $\times$ 4.26 visual degrees (vd)) for large stimuli, 5.5 $\times$ 3.9 cm (4.04 $\times$ 2.86 vd) for medium stimuli, and 2.7 $\times$ 1.9 cm (1.98 $\times$ 1.40 vd) for small stimuli. The stimuli had a resolution of 261 $\times$ 353, 172 $\times$ 232, and 86 $\times$ 116 pixels, respectively. Participants saw only one expression per identity and size. In addition, scrambled versions of a subset of faces were created by shuffling squares of pixels from the face area and adding a mask to account for edge effects. Due to the different lighting, the images differed in luminance and contrast between the databases, which we could not fully reduce without creating visible artifacts. Also, scrambling made the stimuli slightly brighter, although scrambling was restricted to the face region, probably due to masking and edge effects (see luminance measures in the Supplementary Information). Due to this confounding factor, it is important to interpret the effects of expression manipulation, i.e., comparing artificial expressions with natural ones, accordingly.

#### Creating fake expressions

The main reason for creating these artificial faces was to have a larger stimulus set of different identities with comparable attributes, although we were also interested in whether participants would be sensitive to real and manipulated facial expressions. Face databases commonly used in neurophysiological research, which include facial expressions of emotion, often contain only a limited number of identities and are difficult to merge due to large differences in brightness, color, and contrast (see Supplementary Information). In our study, the inclusion of a large number of individual faces, each with different expressions, was required to avoid memory and transfer effects that may arise from the viewing of identical faces of different sizes. The GFD is a high-quality database of faces with many different identities, but only neutral expressions. We have developed an in-house solution for generating happy, angry, and neutral expressions from these previously non-expressive faces, motivated by restrictions on data sharing of the GFD, as well as the sensitivity of facial information. Moreover, most commercial tools were susceptible for artifacts, such as homogenizing facial features when hair and image background were removed. An illustration of the procedure can be found in the Supplementary Information.

In the first step, we used *OpenFace landmark detections*^50^ to automatically align, rotate, and scale faces in an image. The images were then cropped and resized to 128 $\times$ 128 pixels for use in the GAN. Expression manipulations were performed using the publicly available GAN *Ganimation-replicate*^51^, which includes the pre-trained model *EmotionNet* and allows to adjust the intensities of action units (AUs^52,53^). Since the resulting images had a resolution of 128 $\times$ 128 pixels and contained facial distortions, we used another GAN dedicated to the restoration and upscaling of small images containing faces and low-quality images, *GFPGAN*^54^. Although important identity information in the face was preserved, the faces appeared slightly posterized and “glossy”. Therefore, we also resized faces showing real expressions to 128 $\times$ 128 pixels and upscaled them with GFPGAN to minimize any differences caused by image restoration.

Subsequently, the images were scanned for detecting strong artifacts or distortions, and a subset of identities and expressions was selected. We excluded extra-facial features from all face stimuli using an oval mask and grayscale conversion, followed by normalization. To create scrambled versions of images, we used *scrambpy* (v0.5.0, *GitHub—Snekbeater/Scrambpy*). We scrambled only chunks of pixels within the face area to avoid including pixels from the image background and to keep the amount of light and dark pixels constant while still being distinguishable from the background. Finally, we resized the images to the respective small, medium, and large presentation sizes that were used in our study.

### Randomization

The stimulus set included 90 different face identities and 36 scrambled images of three sizes each. To ensure an equal number of images per size and to prevent the same image from being presented in different sizes, we pseudorandomized the size of each face and scrambled stimulus for each participant at the beginning of the session. Participants completed a total of 882 trials in seven blocks of 126 images each (90 faces and 36 scrambled), with the order of the images shuffled in each block.

### Procedure

The study was conducted in accordance with the Declaration of Helsinki and approved by the local ethics committee of the Institute of Psychology at the University of Göttingen. Participants were fully informed of the study’s procedures, including all study phases, compensation, and approximate time required, at both enrollment and the beginning of the experimental session.

**Figure 1:**
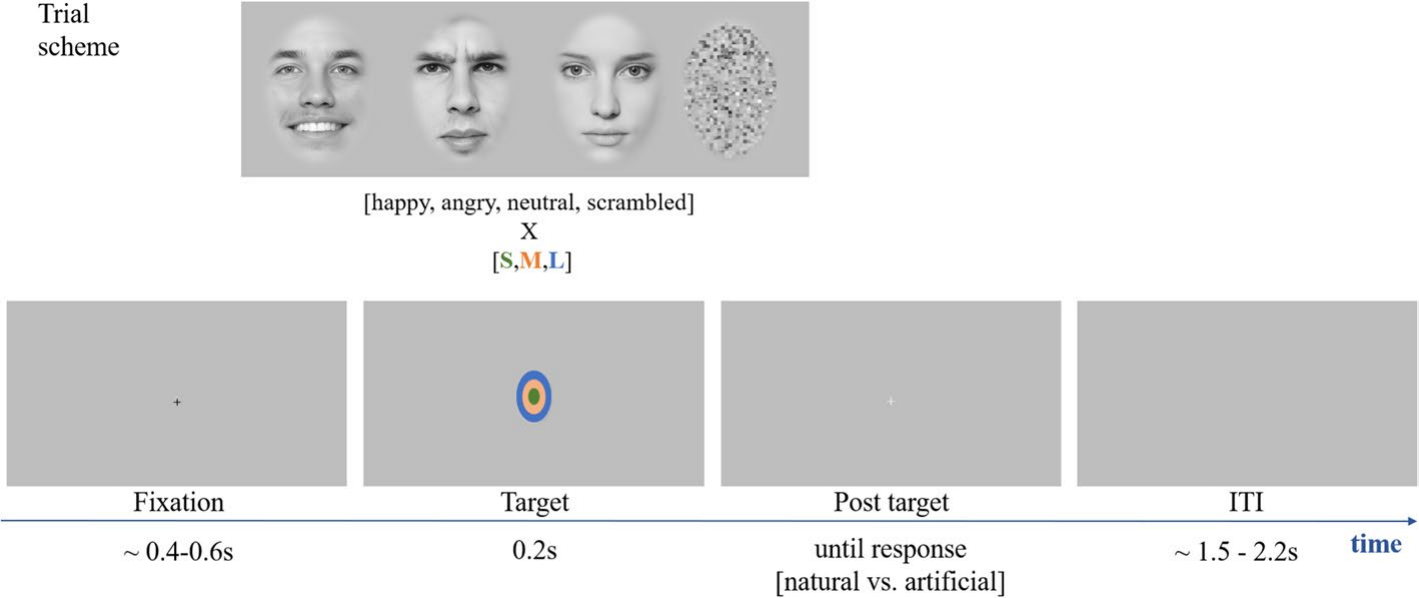
Procedure of the naturalness-classification task of the facial expression. *Notes*: After a black fixation cross with variable duration, an individual face or scrambled stimulus was presented in the center of the screen. The different sizes in which stimuli could be presented are indicated as the colored ovals (only for illustrational purposes). Size ratios between ovals and the box correspond to the presented stimuli and the display size of the monitor. The size of the fixation crosses were increased in this figure for visibility. All exemplary faces show manipulated, i.e., fake expressions. Photographs are from the Göttingen Faces Database^48^. Permission obtained.

The participants’ sociodemographic information was collected at enrollment to assess their eligibility for the experiment. Written informed consent was obtained at the beginning of the experimental session. Participants were seated in a dimly lit, electrically shielded room, in front of a computer screen (BenQ XL2411Z, 24″/27″, 1920 × 1080 px; 144 Hz) at an approximate viewing distance of 78 cm. To prevent head movements, participants positioned their chins on a height-adjustable chin rest. The experiment was run using functions of PsychoPy^55^ in Python (v2.7). PyGaze^56^ (v0.6.5) was used to communicate with the eye-tracker. After a 9-point calibration of the eye tracker, participants were instructed about the task and performed four example trials with feedback on the correctness of their responses. Participants were informed that they would see faces with happy, neutral, or angry expressions, some of which were manipulated, while others were real. We explained that the manipulation was performed by a “neural network” that could change the facial expression. In other words, the individual depicted in an image may have displayed a different expression at the time the picture was taken. The participant’s task was to indicate whether the presented face showed a natural or an artificial expression (go conditions). When a scrambled image was presented, no response was allowed (no-go condition). There was no information on the number of manipulated expressions (50%) or on how to detect them. After ensuring that the task was understood, participants started the experiment. During the main task, no feedback on correct or incorrect answers was given, but participants could choose to see their performance at the end of the task. Behavioral data (reaction times, hit rate, eye gaze) and psychophysiological data (electroencephalography (EEG), eye gaze and pupil size) were collected during the main experiment. An illustration of the procedure and example stimuli can be found in Fig. 1.

Each trial began with a black fixation cross presented at the center of the screen for 0.4–0.6 s (uniformly distributed). Then, a face or scrambled stimulus was presented for 0.2 s, replaced by a white fixation cross displayed until a response was given, or for 0.2 s in no-go trials. The next trial started after an inter-trial interval of 1.5–2.2 s (uniformly distributed), with a blank screen. During the main part of the experiment, there were breaks to recover after every 100 trials. Before resuming the task, the eye tracker was recalibrated through a 1-point calibration/drift correction. The experiment could be paused and resumed, for instance to fix noisy electrodes. Finally, participants received a debriefing which included information on the main aims and background of the study (presented on the computer screen) and were invited to ask the experimenters any additional questions.

### EEG recording and processing

EEG data were recorded at a sampling rate of 512 Hz and a bandwidth of 102.4 Hz, using the software ActiView and a Biosemi ActiveTwoEEG AD-Box with 128 active electrodes (AgAgCl) mounted in an electrode cap (Easy Cap™). For electrode placement, we used the ABC radial layout. In addition, six external electrodes were located on the left and right mastoids, and one each below and beside the both eyes. The common mode sense (CMS) active electrode and the driven right leg (DLR) passive electrode were used as reference electrodes. Based on previous studies in the lab^35,44^ and consistent with typical ERP studies on emotional face processing^4^, we determined the time windows and electrode regions (ROIs) for the ERP components of interest, which were confirmed by pilot data (*N* = 4) that were not included in the analysis. The following preregistered ERPs were extracted for the visual (face-locked) components: **P1**: Peak amplitudes and peak latency, 80–120 ms; occipital electrode cluster: A8, A9, A10, A15 (O1), A16, A17, A28 (O2), A29, A30, B5, B6, B7; **N170**: Peak amplitudes and peak latency, 130–200 ms; occipitotemporal electrode cluster: D32 (P9), A10 (PO7), A11, A12, B10 (P10), B7 (PO8), B8, B9; **EPN**: mean amplitudes, 250–300 ms; occipito-temporal cluster: A10 (PO7), A11, A12, A14, A15 (O1), D32 (P9), A24, A25, A26, A27, A28 (O2), B7 (PO8), B8, B9, B10(P10); **LPC**: mean amplitudes, 400–600 ms; occipito-parietal electrode cluster: A4, A19 (Pz), A20, A21 (POz), A5, A32, A18, A31, A17 (PO3), A30 (PO4). Peak amplitudes were calculated based on the condition-averaged ROI channel maximum (P1) or minimum (N170) value of the respective ROI time window. In addition (not preregistered), we included the mean amplitudes for the ROI time windows of the P1 and N170 to allow for comparisons between studies.

The continuous EEG was preprocessed offline using functions from EEGLAB^57^ (v2019.0) in MATLAB (2018). Event triggers were shifted by a constant of 26 ms to account for the systematic delay of stimulus appearance on the monitor. The continuous data were re-referenced to the whole-head average, excluding external electrodes, and high-pass filtered at 0.01 Hz. The 50 Hz line noise was reduced using “CleanLine”^58^ (v1.04), an EEGLAB plug-in. The data were then epoched from − 500 to 1000 ms around the stimulus onset and corrected to a 200 ms pre-stimulus baseline. For artifact correction, we performed Independent Component Analysis (ICA) on a 1 Hz high-pass filtered version of the data and applied ICA weights to the original 0.01 Hz filtered data. Independent components were removed if they were classified as eye, muscle, or channel noise components with > 90% probability using “ICLabel”^59^ (v1.2.4). We interpolated the remaining channels affected by noise. We performed trial-wise rejections of epochs trimmed to − 200 to 1000 ms, rejecting amplitudes greater than − 100/100 $\mathrm{\mu V}$ during − 200 and 600 ms (avr. 5.3%), steep amplitude changes (> 100 $\mathrm{\mu V}$ within the epoch; avr. 5.9%), and improbable activation (deviation > 3 from the mean distribution for each time point; 0%). In the final sample of participants, these artifact rejection methods caused an average exclusion rate of 7.8% (range 0.1–38.2%) of trials. We measured pupil size and gaze to detect blinks and fixation deviations from the target with a desktop-mounted eye tracker (EyeLink 1000 CL 1—AAD01, SR Research; v4.56). ERP trials that contained blinks in the baseline and time windows of interest were excluded from the analyses.

### Statistical analysis

All statistical analyses were performed in R (v4.0). (Generalized) linear mixed models were used to analyze behavioral and ERP data, and the maximum likelihood (ML) estimator was used to estimate parameters. Each response variable was analyzed using a separate model. Statistical significance was inferred using likelihood ratio tests (LRT), which compared a model with the predictor of interest against a reduced model without it. For significant LRTs, we reported post-hoc contrasts for the difference between factor levels. The conventional significance level $\alpha$ = 0.05 (two-tailed) was used, and post-hoc tests were Šidák -corrected to adjust for multiple comparisons. We checked the models for potential variance inflation and the model residuals for potential model misspecification. Tables of results for all models, including regression coefficients $\beta$, standard errors (SE), 95% nonparametric bootstrapped confidence intervals (CI), and coefficient stability (leave-one(participant)-out), are included in the Supplementary Information.

We preregistered two models for each ERP component of interest: (a) a model including stimulus size (with three levels) $\times$ Emotion_+Scram_ (with four levels: scrambled (reference), happy, angry, neutral), and (b) a model including stimulus size (with three levels) $\times$ Face_+Scram_ (with two levels: scrambled (reference), intact faces). However, since the ERPs of scrambled stimuli were very different from all face categories, we dropped this factor in model (a) to avoid confusing a significant effect of the “Emotion_+Scram_” factor with differences between emotion levels, and because this factor is already included in (b). The originally preregistered model results, the results of extended models including the expression manipulation (real/fake) as a predictor, and a discussion of stimulus size and face intactness can be found in the Supplementary Information.

Behavioral analysis was applied only to the intact stimuli, as the scrambled versions were considered as “no-go” stimuli. We included correct and incorrect responses and trimmed RTs to an upper threshold of 5000 ms. For each participant and condition separately, a skewness-adjusted boxplot^60^ method was applied to exclude extreme values (“adjbox” function from the R package “robustbase”^61^). The RT model estimation was based on the mean responses per condition and participant. We used a linear mixed model to estimate the RT effects of emotion, stimulus size, and their interaction, and included a random intercept for participant ID. The analysis of response accuracy was exploratory and not preregistered. We conducted a mixed logistic regression with emotion, stimulus size, and their interaction as fixed effects. Random slopes of emotion and stimulus size were included, in addition to the random intercept for participant ID, to reduce overdispersion of the model. Real-or-artificial decisions to the faces were further analyzed using a generalized linear mixed probit model as a signal detection method, in which the signal would correspond to the presence of a real expression. This allows separate modeling of the response criterion (c) and discriminability index (d′). The model specification and all estimates are included in the Supplementary Information.

### Ethics approval

The study was conducted in accordance with the Declaration of Helsinki and approved by the local Ethics committee of the Institute of Psychology at the University of Göttingen. Consent to participate Informed consent was obtained from all individual participants included in the study.

## Results

### Behavioral outcomes

**Figure 2:**
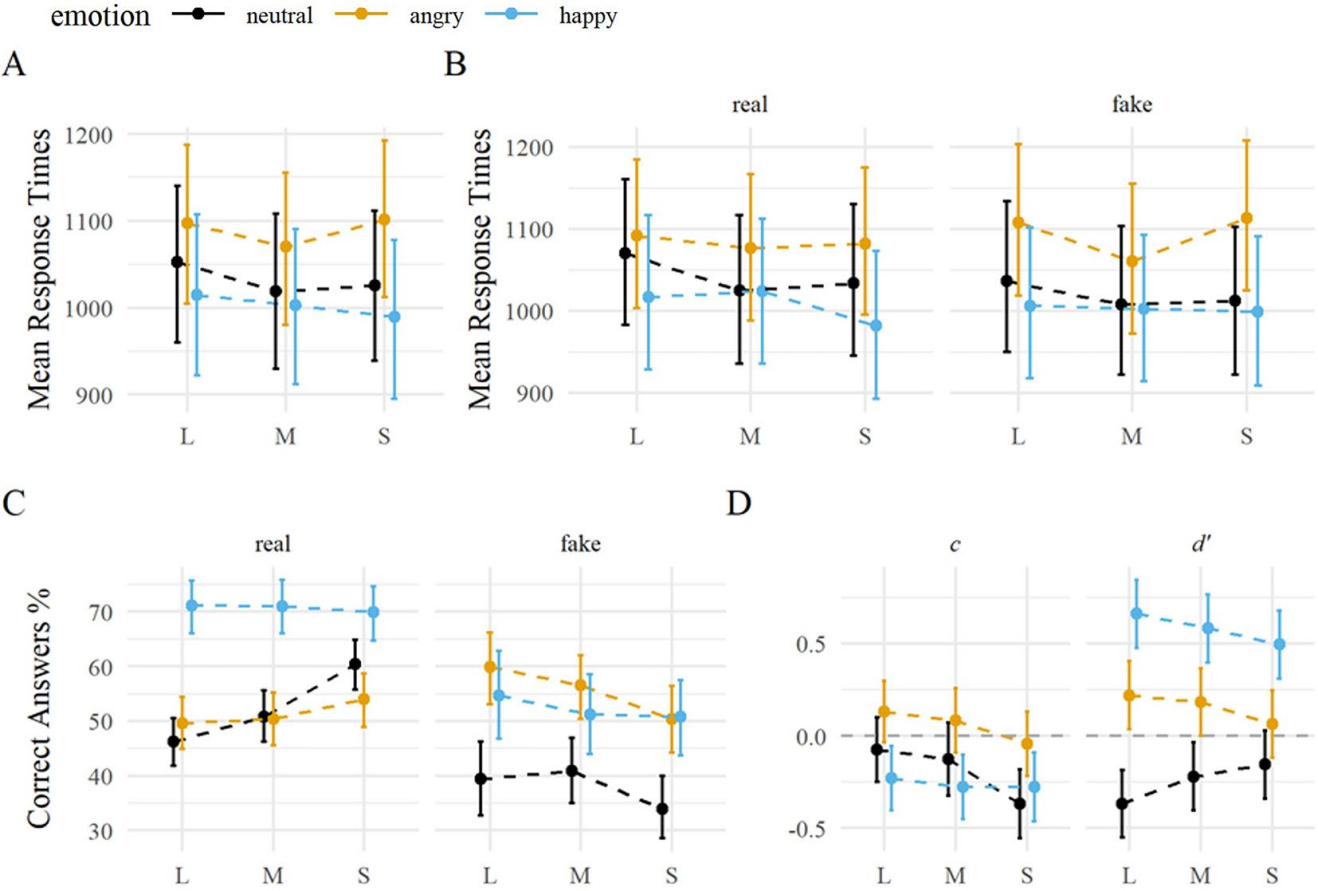
Accuracy and response times as a function of emotion, stimulus size, and expression manipulation in the naturalness classification task. *Notes* (**A**) Response times in milliseconds. Mean values were averaged per emotion, stimulus size, and (**B**) expression manipulation over participants. (**C**) Probability of correct answers in percent, separately for manipulation. (**D**) Estimated response criterion *c* and discriminability index *d’* (signal detection theory; signal in this context refers to the presence of a real expression). Error bars depict the 95% CI.

Response times were modulated by emotion (${\chi }^{2}$(2) = 85.57, *p* < 0.001) with happy faces being responded to fastest (est_hap_ = 1002 ms), followed by neutral (est_neu_ = 1032 ms) and angry faces (est_ang_ = 1090 ms). All pairwise comparisons between emotion levels were significant on *p* < 0.01 (adjusted) . There was also an effect of stimulus size (${\chi }^{2}$(2) = 7.37, *p* = 0.025), with large stimuli being significantly slower responded to than medium-sized stimuli (diff_L–M_ = 24.14, *p* = 0.025), but not between large and small (diff_L–S_ = 15.79, *p* = 0.230) or medium and small (diff_M–S_ = − 8.35, *p* = 0.737). The interaction between stimulus size and emotion was not significant (${\chi }^{2}$(4) = 5.25, *p* = 0.263). An extended model showed no significant main effect of expression manipulation nor interaction with other variables (all *ps* > 0.1), see Fig. 2.

There was a main effect of emotion on **accuracy** (${\chi }^{2}$(2) = 26.90, *p* < 0.001), with a higher accuracy for happy compared to angry faces (*OR*_hap/ang_ = 1.39, *p* =  < 0.001), and for angry compared to neutral faces (*OR*_ang/neu_ = 1.36, *p* =  < 0.001). Stimulus size did not affect accuracy (${\chi }^{2}$(2) = 0.13, *p* = 0.938), but there was a significant emotion $\times$ stimulus size interaction (${\chi }^{2}$(4) = 16.17, *p* = 0.003). Within a stimulus size, accuracy differences were significant for all emotion levels, but only reached trend level for the difference between small angry and small neutral faces (*OR*_s.ang/s.neu_ = 1.2, *p* = 0.096). The signal detection model showed both effects of emotion and stimulus size on response criterion (*c*) and discriminability (*d’*). The response criterion was significantly below zero for happy faces at all stimulus sizes (all *c*_hap_ <  = − 0.23, all *p* <  = 0.010) and for small neutral faces (*c*_S_neu_ = − 0.37, *p* < 0.001), which had the overall lowest criterion. The most positive (but insignificant) criterion was found for large angry faces (*c*_L_ang_ = 0.13, *p* = 0.128). There was an overall tendency for a decreasing criterion with decreasing stimulus size, with a shift in criterion most pronounced for neutral faces (*c*_L_neu_ − *c*_S_neu_ = 0.29, *p* < 0.001) and least pronounced for happy faces (*c*_L_hap_ − *c*_S_hap_ = 0.05, *p* = 0.713). Discriminability differed between expressions and was greatest for happy faces (all *d’* >  = 0.49, all *p* < 0.001). There was a tendency for discriminability to increase with stimulus size for happy and angry faces (*d*′_L_hap_ − *d*′_S_hap_ = 0.168, *p* = 0.063; *d*′_L_ang_ − *d*′_S_ang_ = 0.156, *p* = 0.079). In contrast, large neutral faces showed negative discriminability indices (*d*′_L_neu_ = − 0.37, *p* < 0.001), indicating a higher probability for artificial faces than for real faces to be classified as real, see Fig. 2.

### ERP results

#### P1

Mean amplitudes were not modulated by emotion (${\chi }^{2}$(2) = 3.62, *p* = 0.163) but by stimulus size (${\chi }^{2}$(2) = 8.83, *p* = 0.012), with larger amplitudes for medium and large faces compared to small faces (diff_M–S_ = 0.31, *p* = 0.028; diff_L–S_ = 0.30, *p* = 0.038), and no difference between medium and large faces (diff_M–L_ = 0.01, *p* = 0.999). There was no interaction between emotion and stimulus size (${\chi }^{2}$(4) = 2.50, *p* = 0.645). When including expression manipulation in the model, there was a trend for a main effect of manipulation (${\chi }^{2}$(1) = 3.31, *p* = 0.069) and a trend for a three-way interaction of emotion, stimulus size and manipulation (${\chi }^{2}$(4) = 7.93, *p* = 0.094), which was significant for the P1 peak amplitudes (see below).

Peak amplitudes were also not modulated by emotion (${\chi }^{2}$(2) = 4.14, *p* = 0.126) but by stimulus size (${\chi }^{2}$(2) = 42.68, *p* < 0.001), with larger mean amplitudes for medium and large faces than for small faces (diff_M–S_ = 0.65, *p* < 0.001; diff_L–S_ = 0.83, *p* < 0.001). Medium and large faces did not differ significantly (diff_M–L_ = − 0.18, *p* = 0.413), and the interaction between emotion and stimulus size was not significant (${\chi }^{2}$(4) = 0.82, *p* = 0.935). When including expression manipulation in the model, the three-way interaction of emotion, stimulus size and manipulation was significant (${\chi }^{2}$(4) = 10.32, *p* = 0.035), mainly driven by differences between fake happy and angry expressions in medium sized stimuli (diff_hap-ang_ = − 1.01, *p* = 0.009).

**Figure 3:**
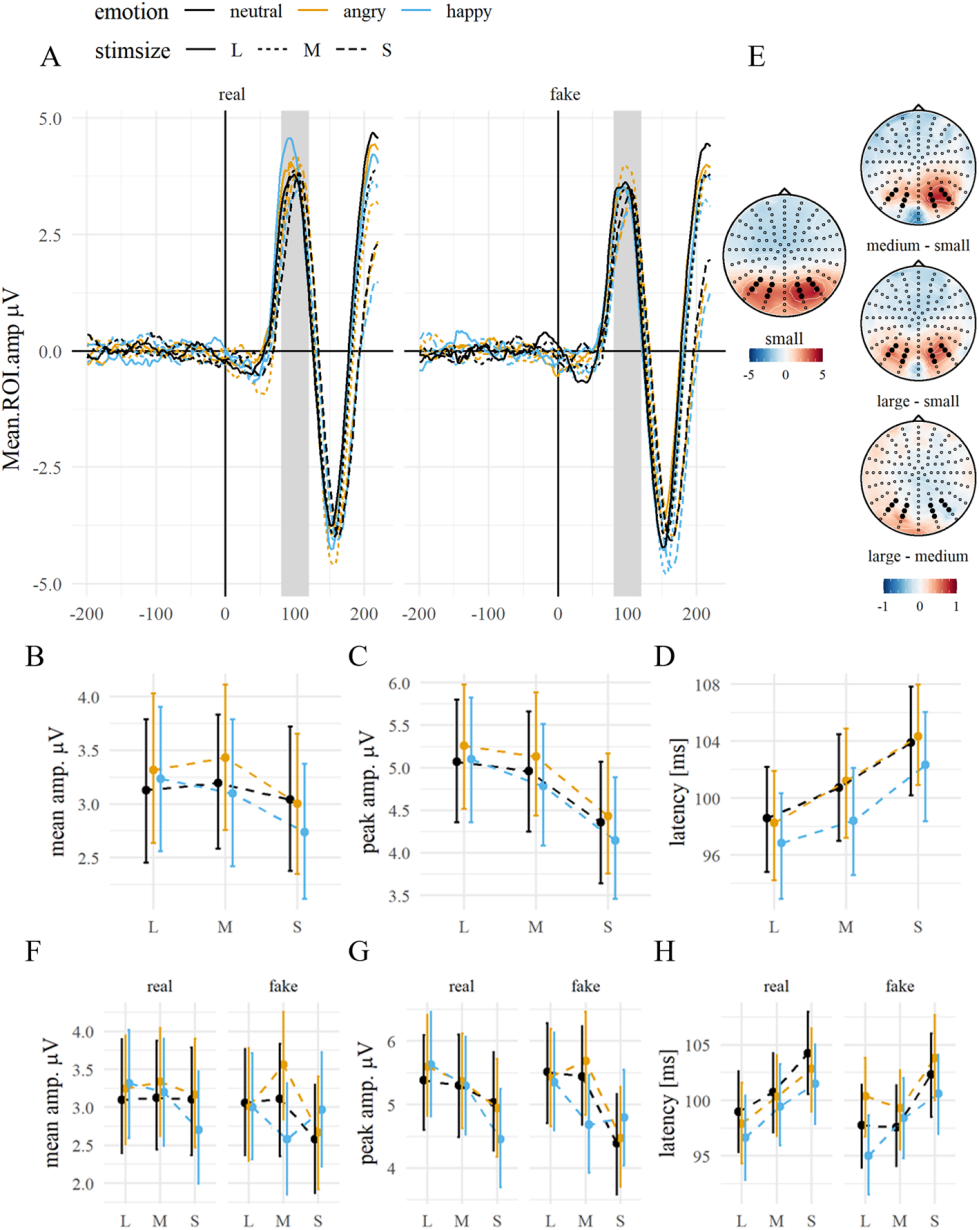
P1 by emotion, stimulus size and expression manipulation. *Notes:* (**A**) Grand average ERP time series of the averaged ROI channels. The highlighted area displays the ROI time window. (**B**) Grand averages of the ROI mean amplitudes and (**C**) peak amplitudes, and (**D**) peak latencies, contrasted for stimulus sizes and all emotion conditions. Error bars indicate the 95% CI. (**E**) Topographies of the ERP distribution for small faces and pairwise differences of size levels, averaged over emotion levels. The highlighted channels depict the ROI channels. (**F**–**H**) show the respective averages split by expression manipulation.

Peak latency showed a main effect of emotion (${\chi }^{2}$(2) = 6.94, *p* = 0.031). However, post-hoc contrasts showed only a trend for happy faces, eliciting P1 with shorter latencies compared to neutral and angry faces (diff_hap-ang_ = − 2.08, *p* = 0.054; diff_hap-neu_ = − 1.89, *p* = 0.095). There was a main effect of stimulus size (${\chi }^{2}$(2) = 40.24, *p* < 0.001), with shorter latencies for large compared to medium faces (diff_L–M_ = − 2.23, *p* = 0.035) and for medium compared to small faces (diff_M–S_ = − 3.42, *p* =  < 0.001). The interaction between emotion and stimulus size was not significant (${\chi }^{2}$(4) = 0.52, *p* = 0.971), see Fig. 3. For the extended model, there was only a trend for an interaction between emotion and manipulation (${\chi }^{2}$(2) = 5.08, *p* = 0.079), driven by differences between real happy and neutral expressions (diff_hap-neu_ = − 3.17, *p* = 0.003).

#### N170

**Figure 4:**
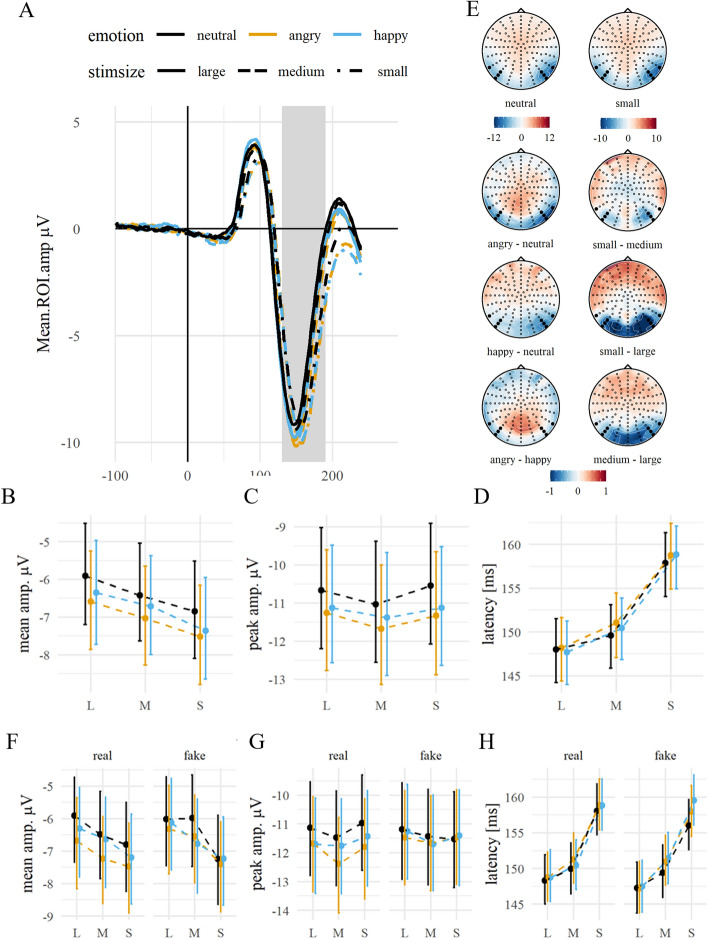
N170 by emotion and stimulus size. *Notes:* (**A**) Grand average ERP time series of the averaged ROI channels. The highlighted area displays the ROI time window. (**B**) Grand averages of the ROI mean amplitudes and (**C**) peak amplitudes, and (**D**) peak latencies, contrasted for stimulus sizes and all emotion conditions. Error bars indicate the 95% CI. (**E**) Topographies of the ERP distributions. The left column shows the main effect of emotion, with the topography for neutral expressions, and the pairwise differences between emotion levels (all averaged over sizes). The right column shows the main effect of size, with the topography of small faces and pairwise differences of size levels (all averaged over emotion levels). The highlighted channels depict the ROI channels. (**F**–**H**) show the respective averages split by expression manipulation.

Mean amplitudes were modulated by both emotion (${\chi }^{2}$(2) = 19.78, *p* < 0.001) and stimulus size (${\chi }^{2}$(2) = 40.72, *p* < 0.001), but the interaction was not significant (${\chi }^{2}$(4) = 0.46, *p* = 0.977). For all stimulus sizes, N170 mean amplitudes were increased for happy and angry compared to neutral faces (diff_hap-neu_ = − 0.41, *p* = 0.016; diff_ang-neu_ = − 0.65, *p* < 0.001). Mean N170 amplitudes were negatively related to stimulus size, with increasing amplitudes from large to medium to small faces (diff_S–M_ = − 0.52, *p* = 0.001; diff_M–L_ = − 0.44, *p* = 0.010). Peak amplitudes were also modulated by both emotion (${\chi }^{2}$(2) = 30.63, *p* < 0.001) and stimulus size (${\chi }^{2}$(2) = 11.36, *p* = 0.003), but not by their interaction (${\chi }^{2}$(4) = 0.93, *p* = 0.920). They were significantly larger in response to emotional than neutral faces (diff_hap-neu_ = − 0.46, *p* < 0.001; diff_ang-neu_ = − 0.67, *p* < 0.001), and for medium faces compared to small (diff_M–S_ = − 0.36, *p* = 0.010) and to large faces (diff_M–L_ = − 0.35, *p* = 0.014). There was no significant peak amplitude difference in response to large and small faces (diff_L–S_ = − 0.02, *p* = 0.999). N170 peak latency was not modulated by emotion (${\chi }^{2}$(2) = 3.66, *p* = 0.160) but by stimulus size (${\chi }^{2}$(2) = 337.03, *p* < 0.001), with shorter latencies for large faces compared to medium (diff_L–M_ = − 2.41, *p* < 0.001) and for medium compared to small faces (diff_M–S_ = − 8.12, *p* < 0.001). The interaction between emotion and stimulus size was not significant (${\chi }^{2}$(4) = 2.21, *p* = 0.697), see Fig. 4. The extended model showed no interaction with expression manipulation on mean or peak amplitudes or peak latencies (all *p*s >  = 0.05), although there was a trend for a main effect on the N170 peak latency (${\chi }^{2}$(1) = 3.34, *p* = 0.068), driven by a tendency for real expressions to peak more slowly (*β*_real_ = 0.33, *CI* = [− 0.03; 0.68].

#### EPN

**Figure 5:**
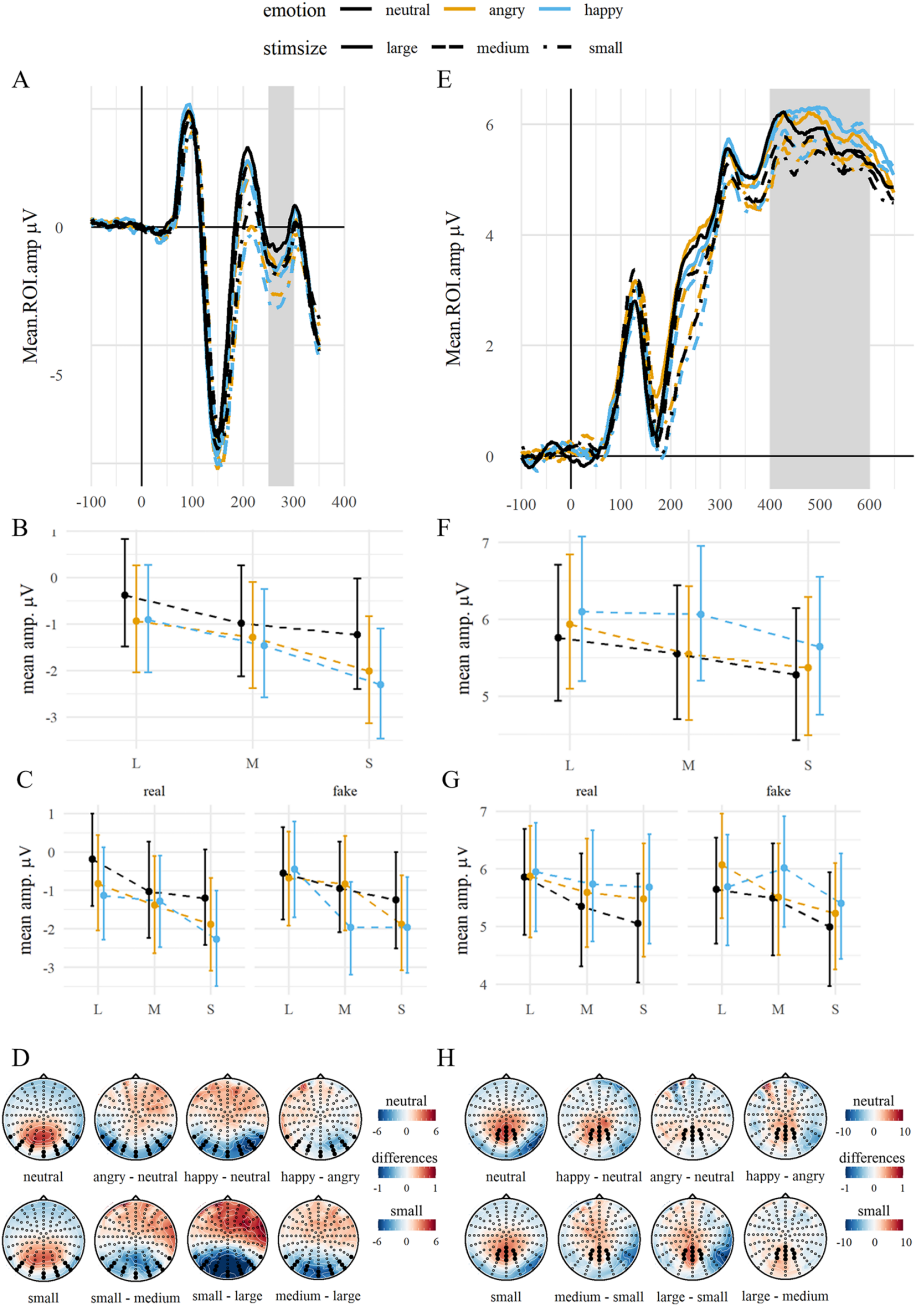
EPN and LPC by emotion and stimulus size. *Notes:* (**A**) EPN and (**E**) LPC: Grand average ERP time series of the averaged ROI channels. The highlighted area displays the ROI time window. (**B**) EPN and (**F**) LPC: Grand averages of the ROI mean amplitudes contrasted for stimulus sizes and all emotion conditions. Error bars indicate the 95% CI. (**C**) EPN and (**G**) LPC: The respective averages split by expression manipulation. (**D**) EPN and (**H**) LPC: Topographies of the ERP distributions. The top row shows the main effect of emotion, with the topography for neutral expressions, and the pairwise differences between emotion levels (averaged over all sizes). The bottom row shows the main effect of size, with the topography of small faces and pairwise differences of size levels (averaged over all emotion levels). The highlighted channels depict the ROI channels.

There was a main effect of emotion on EPN amplitudes (${\chi }^{2}$(2) = 29.42, *p* < 0.001), with increased negativities for happy and angry faces (diff_hap-neu_ = − 0.69, *p* < 0.001; diff_ang-neu_ = − 0.54, *p* < 0.001) compared to neutral faces and no differences between happy and angry faces (diff_hap-ang_ = − 0.15, *p* = 0.615). Stimulus size also modulated EPN amplitudes (${\chi }^{2}$(2) = 64.14, *p* < 0.001) and was negatively related to EPN amplitudes with the largest amplitudes for small faces, followed by medium and large stimuli (diff_M–S_ = 0.60, *p* < 0.001; diff_L–M_ = 0.50, *p* < 0.001). There was no significant interaction between emotion and stimulus size (${\chi }^{2}$(4) = 5.00, *p* = 0.287), see Fig. 5. The extended model showed a trend for a three-way-interaction with expression manipulation (${\chi }^{2}$(4) = 9.28, *p* = 0.055). Within small stimuli, only real expressions differed significantly between happy and neutral faces (diff_hap-neu_ = − 1.07, *p* = 0.009) but emotion contrasts were not significant in fake expressions (all ps >  = 0.05). Similarly, within large stimuli, only real happy and neutral expressions differed significantly (diff_hap-neu_ = − 0.94, *p* = 0.025). In contrast, within medium sized stimuli, there was only a difference between fake happy and neutral and happy and angry expressions (diff_hap-neu_ = − 1.01, *p* = 0.017; diff_hap-ang_ = − 1.13, *p* = 0.007).

#### LPC

LPC amplitudes were modulated by emotion (${\chi }^{2}$(2) = 17.32, *p* < 0.001) and stimulus size (${\chi }^{2}$(2) = 23.90, *p* < 0.001), whereas their interaction was not significant (${\chi }^{2}$(4) = 2.20, *p* = 0.700). Happy faces elicited larger amplitudes compared to neutral (diff_hap-neu_ = 0.41, *p* < 0.001) and angry faces (diff_hap-ang_ = 0.32, *p* = 0.006). Amplitudes between angry and neutral faces did not differ (diff_ang-neu_ = 0.09, *p* = 0.778). Amplitudes for small stimuli differed from medium (diff_M–S_ = 0.29, *p* = 0.016) and large (diff_L–S_ = 0.50, *p* < 0.001), whereas they did not differ between medium and large (diff_M–L_ = − 0.21, *p* = 0.107), see Fig. 5. The extended model showed no main effect of or interaction with expression manipulation (all *p*s >  = 0.05).

## Discussion

This study investigated the potential influence of stimulus size on the perception of emotional expressions in faces. We presented a large set of faces with angry, happy, or neutral expressions in three different sizes and systematically tested size, emotion, and interaction effects on early, mid-latency, and long-latency event-related potentials (ERPs). The experimental task was to judge the naturalness of briefly presented authentic or altered facial expressions, thereby increasing the drive to carefully analyze the faces without explicitly identifying the emotion conveyed. We expected general effects of size and emotion on ERP components, while specifically investigating whether emotion effects would be amplified with increasing face size. Behavioral accuracy indicates that emotional expression and stimulus size interact to impact the processing of real and artificial expressions. However, the electrophysiological responses suggested overall additive effects rather than a systematic increase in emotion effects with increasing stimulus size^cf.^^24,25^. While stimulus size affected all ERP components, main effects of emotion were observed for the N170, the EPN, and the LPC. To also account for potential differences in stimuli based on our manipulation, we tested for the main and interaction effects of expression manipulation.

Consistent with previous findings^37,38^, larger stimuli elicited P1 components with enhanced amplitudes and shorter latencies. In contrast, the emotional expression of the face did not impact P1 amplitudes, although there was a tendency towards shorter peak latencies for faces with happy, particularly real, expressions. The only significant emotion-related difference in P1 amplitudes was observed between fake happy and angry expressions, limited to medium-sized stimuli. It is possible that this effect results from a combination of the specific size and the stimulus configuration differences. A replication with a larger, independent sample and more gradual size steps is necessary to model the underlying, potentially non-linear, function between size and configuration in faces. Generally, increasing stimulus sizes did not significantly enhance the effects of emotion. This contradicts the findings of larger P1 amplitudes for looming angry faces^28^. Although it also has been reported for later ERPs (e.g., for looming threat-related animals^62^), looming might enhance earlier emotion-sensitive ERPs, especially those elicited by faces, and prove more effective than the static images used in the present study. Similarly, enhanced emotion effects on later ERPs have been shown for dynamic facial expressions^63^.

The earliest significant main effect of emotion in our study manifested in the N170 component. As hypothesized, negative and, to a lesser degree, positive expressions evoked enhanced negative amplitudes compared to neutral expressions (descriptively more pronounced for real expressions), corroborating reported N170 effects of happy and threat-related expressions^4,64,65^. Because N170 peak latencies differed across sizes, possibly as a continuation of earlier processing, size effects on mean amplitudes must be interpreted accordingly. Notably, N170 peak amplitudes were most pronounced for medium-sized faces across various expression levels, suggesting that emotion effects on the N170 were not generally amplified by larger stimulus sizes. Possibly, the greater activation might be attributed to the spatial frequency configuration of medium-sized faces employed in this study. In general, the N170 does not seem to be size-invariant. While many face-selective neurons in the superior temporal sulcus of macaques show size-invariance, a minority of these neurons demonstrate unique responses to retinal angle and absolute size. It has been proposed that both these factors contribute to size-invariant face recognition, including descriptors of plausible absolute face sizes^66^. Similarly, most face-selective neural responses in the temporal and occipital lobes of humans occur before the typical N170 range (approximately at 100 ms^67^), irrespective of the scaling of the faces. However, some evidence demonstrates that activation in the fusiform face area is affected by size variations, particularly when comparing large ranges of sizes^68^. N170 amplitudes for faces have been shown to be modulated not only by size but also by resolution^32^. Whether the N170 size effect could have been carried over by earlier processes or reflect parallel feedback loops and back-projections that are involved in memory, emotion, and action processes remains a question for future research.

As hypothesized, emotion effects also extended to the EPN component (for a discussion of the functional distinction between the N170 and the EPN^6^). Consistent with previous studies^36,64,69^, the EPN was modulated by both happy and angry faces and did not statistically differ between emotional expressions^cf.^^70,71^ when averaged over real and fake stimuli. There was a main effect of size on the EPN, but contrary to our prediction and the expected relevance effect, small stimuli resulted in increased negative amplitudes^cf.^^24^, and descriptively, the difference between emotional and neutral expressions (in real expressions) was also largest for small stimuli, although the interaction was not significant. When considering real and fake expressions separately, there was no gradual increase in amplitude for emotional compared to neutral expressions as a function of size, neither in real nor fake expressions.

As predicted, the LPC was modulated by both stimulus size and emotion largely independently (similar to related studies^24,25^). Across emotion levels, increased LPC amplitudes were found for medium and large compared to small faces, indicating sustained attention as a function of stimulus size. When collapsing over real and fake expressions, across all stimulus sizes, LPC amplitudes were largest for happy expressions and, descriptively, amplitudes were also larger for angry faces than for neutral faces. However, the difference between neutral and angry faces was not significant, which contradicts our initial prediction of a general negativity bias towards anger stimuli^42,72^.

Since the predicted interactions between emotion and size (also not when considering real and fake expressions separately) were not observed in the ERPs, we performed power simulations on the emotion × stimulus size interaction effects of each ERP component (for the statistical models and estimates see the Supplementary Information). These analyses revealed a power of 10% for detecting the estimated effect of *f*^2^ = 0.003 for P1 peak amplitudes, 11.50% (*f*^2^ = 0.003) for N170 peak amplitudes, 42.50% (*f*^2^ = 0.016) for the EPN, and 21% (*f*^2^ = 0.007) for the LPC. Evidently, the observed modulations of emotion effects by size in our study were very small, which indicates their negligible impact for the range of sizes used. For comparison, the sample sizes of the studies that reported interactions between stimulus size and emotion of substantial magnitude (*f*^2^ ≈ 3.050 [*η*^2^_*p*_ = 0.75]*, N* = 16^25^; and *f*^*2*^ ≈ 0.145 [*η*^*2*^_*p*_ = 0.127]*, N* = 25^24^) were considerably smaller than the number of participants included in our study (*N* = 40).

Our findings must be considered in the light of the specific task participants performed on the faces. We selected the naturalness decision task to foster processing of facial expressions without explicitly deciding about their emotional valence. Since emotion effects on later ERPs have been shown to be task-sensitive^6^, we aimed to detect emotion effects when no valence/emotion classification of the expression is required, while prompting deeper processing compared to tasks like passive viewing or gender decisions. The behavioral results of the naturalness classification task revealed both effects of emotional expression and stimulus size on response times, as well as an interaction of emotion and stimulus size on accuracy. As expected, participants were overall more accurate in judging the artificiality of happy expressions and their decisions were also faster for happy than for the other emotion categories. This indicates an overall improvement in the processing of relevant facial features, which may also be reflected in the overall heightened LPC amplitudes for happy faces. Furthermore, angry stimuli slowed response times, similarly to previous research on negative or threatening information where valence detection was not task-relevant^2,25,42,73^. However, it is conceivable that slower responses were partially attributed to task difficulty. When considering both response times and accuracy, angry faces were responded to the slowest, and importantly, discriminability was low and accuracy was overall only slightly above chance (and only significantly above chance for large expressions), suggesting that participants were uncertain whether the angry expressions were genuine or artificial. In contrast, neutral expressions, which have presented a more challenging decision due to the marginal difference from the original, resulted in a negative discriminability and overall accuracy below chance, particularly for large neutral faces, indicating that the artificially created expressions were more likely to be evaluated as natural compared to the unaltered expressions.

Stimulus size affected response times largely independently from expression manipulation, with slower responses for large compared to medium-sized stimuli^cf.^^25^. On average, response times were relatively slow compared to common two-forced-choice tasks, such as gender or valence classification decisions^6,35^. It is possible that participants prioritized accuracy over speed while detecting fake expressions. However, it is unclear whether participants actually took advantage of the higher resolution of facial details in larger stimuli and deliberately processed the faces longer, or whether expressions in small faces were processed more efficiently due to the foveal presentation of relevant facial features. There may be a task-specific optimal retinal stimulus size for information extraction in experimental tasks^37^. However, we suspect that speed measures, particularly for the present task, may be less affected by size because the discriminability of facial expressions appears preserved even in small faces^74,75^. This might be partly explained by the relative size invariance of some face-selective regions that were involved in higher-level processes^67,76,77^ during the naturalness-classification task.

In conclusion, additive effects of emotion and stimulus size were found on face-sensitive ERP components. This suggests that the processing of facial expressions of emotion was relatively size-invariant for faces represented within the fovea^78^. Both the face-sensitive N170 and the EPN components were enhanced in response to happy and angry compared to neutral expressions, which was descriptively more pronounced for real expressions. The largest LPC amplitudes and naturalness classification accuracies were observed for happy faces. In addition, all ERP amplitudes were modulated by stimulus size, with more pronounced effects on early components. Contrary to prior studies indicating boosted effects of stimulus size on the processing of emotional valence in words or visual scenes, our results suggest that even for small faces, the discriminability between facial expressions is sufficient to produce typical emotion effects. This holds true at least within the range of stimulus sizes investigated in this study, for which the largest sizes correspond to real faces roughly at a distance of 180 cm (with European face widths ranging from 11.7 to 13.2 cm;^79^^(p. 39)^. (We thank one of the reviewers for the suggestion to include this information.) Although the inclusion of even larger stimuli could lead to higher arousal and stronger emotion effects^20^, it might also introduce side effects as systematic eye movements^22^, or reflect activations of retinal areas outside the fovea which respond more to low spatial frequencies^74^. Likely, retinal size does not exclusively explain the size effects in our study, but rather seems to interact with spatial frequency, as indicated by the stronger effects of size found for scrambled compared to intact faces, and possibly also by differences between fake and real expressions. The dissociation between face-specific and other object-specific size invariance, and its relation to emotion processing, remains an intriguing subject for future research. Moreover, the induced perception of physical proximity through additional contextual cues, such as depth-cued backgrounds, has been shown to enhance the early processing of anger expressions in looming faces^27,28^. This raises the question of the timing and mechanism of the integration of the physical distance of faces in lower- and higher-level processes. Finally, although the stimuli were homogenized in some of their low-level features, physical variations in emotional expressions were inherent to the stimuli, as identical stimuli cannot express different emotions. However, faces have been shown to elicit emotion-related effects when associated with emotional expressions of the face and voice^44,80,81^. To achieve full control of physical stimulus features across valence categories, future studies could use faces with neutral expressions but associated with emotional relevance and presented at different sizes.

### Supplementary Information

Supplementary Information.

## Data Availability

The conditions of our ethics approval do not permit public archiving of study data. The entire data and stimulus sets will be made available to interested researchers following completion of a data sharing agreement and approval by the local ethics committee. The analysis and experimental code of this study is available upon request from the corresponding author, AZ. The study was preregistered before data collection (https://osf.io/evfks).
