# Supplementary material for: Additive effects of emotional expression and stimulus size on the perception of genuine and artificial facial expressions: an ERP study
Source: Sci Rep. 2024 Mar 6;14:5574. doi: 10.1038/s41598-024-55678-2 (PMC10918072; doi:10.1038/s41598-024-55678-2)
Supplement: Supplementary file 1 — Supplementary Information. [file 41598_2024_55678_MOESM1_ESM.pdf]

Supplementary Information for: Additive effects of emotional expression and stimulus size on the perception of genuine and artificial facial expressions: An ERP study

Annika Ziereis & Anne Schacht

2024/02/19

Contents

Face intactness (faces vs. scrambled stimuli) and stimulus size. 1
Preregistered statistical models 4
Exploratory statistical models (including expression manipulation) 12
Signal detection theory: Analysis of real-fake decisions 17
GLMER Model (response model) to estimate d' and c 17
Creating fake expressions 19
Image properties 20
References 22

Correspondence: Goßlerstr. 14, 37073 Goettingen, Germany Email: Annika.Ziereis@uni-goettingen.de

Face intactness (faces vs. scrambled stimuli) and stimulus size.

ERP results of face intactness (faces vs. scrambled stimuli) and stimulus size are shown in Figure S1.

P1: Mean amplitudes differed between faces and scrambled stimuli ($\chi^2(1)$ = 21.42, p <.001), with smaller amplitudes for faces ($\text{diff}_{\text{face-scrb}}$ = -0.72). There was also an effect of stimulus size ($\chi^2(2)$ = 14.03, p <.001), with differences between small and both medium and large stimuli, but not between medium and large stimuli ($\text{diff}_{\text{M-S}}$ = 0.55, p = .011; $\text{diff}_{\text{L-S}}$ = 0.66, p = .002; $\text{diff}_{\text{M-L}}$ = -0.11, p = .916). The interaction with stimulus size was not significant ($\chi^2(2)$ = 3.80, p = .149).

Peak amplitudes were modulated by intactness of the face ($\chi^2(1)$ = 27.55, p <.001), stimulus size ($\chi^2(2)$ = 44.34, p <.001), and their interaction ($\chi^2(2)$ = 6.84, p = .033). Peak amplitudes differed significantly between face and scrambled stimuli of medium sizes ($\text{diff}_{\text{m.face-m.scrb}}$ = -0.88, p = .002) and large sizes ($\text{diff}_{\text{l.face-l.scrb}}$ = -1.37, p <.001) but not of small sizes ($\text{diff}_{\text{s.face-s.scrb}}$ = -0.34, p = .222).

Peak latency showed only a trend modulation of the face intactness ($\chi^2(1)$ = 2.92, p = .087), with longer latencies for faces compared to scrambled stimuli ($\text{diff}_{\text{face-scrb}}$ = 1.73). There was a main effect of stimulus size ($\chi^2(2)$ = 37.11, p <.001), analogously to the emotion model. No interaction between face intactness and size was present ($\chi^2(2)$ = 3.26, p = .196).

N170: N170 mean amplitudes were only significantly modulated by intactness of the face ($\chi^2(1)$ = 302.79, p <.001), with more pronounced negative amplitudes for intact faces ($\text{diff}_{\text{face-scrb}}$ = -6.89). Neither stimulus size ($\chi^2(2)$ = 1.86, p = .395), nor the interaction with intactness was significant ($\chi^2(2)$ = 3.93, p = .140).

However, peak amplitudes were affected both by intactness ($\chi^2(1)$ = 310.77, p <.001), with more negative amplitudes for intact faces ($\text{diff}_{\text{face-scrb}}$ = -8.13), and by stimulus size ($\chi^2(2)$ = 8.01, p = .018). There was a trend for an interaction ($\chi^2(2)$ = 5.38, p = .068). Post-hoc contrasts showed that the size effect was only apparent within scrambled stimuli, with differences between medium and small ($\text{diff}_{\text{scrb.M-S}}$ = -1.54, p = .010), as well as large and small stimuli ($\text{diff}_{\text{scrb.L-S}}$ = -1.67, p = .005), but not between medium and large stimuli ($\text{diff}_{\text{scrb.M-L}}$ = 0.13, p = .967). Within faces, sizes effects were all insignificant (all p >.05).

Figure S1:
ERPs (P1, N170, EPN, and LPC) of scrambled vs. intact faces by stimulus size.

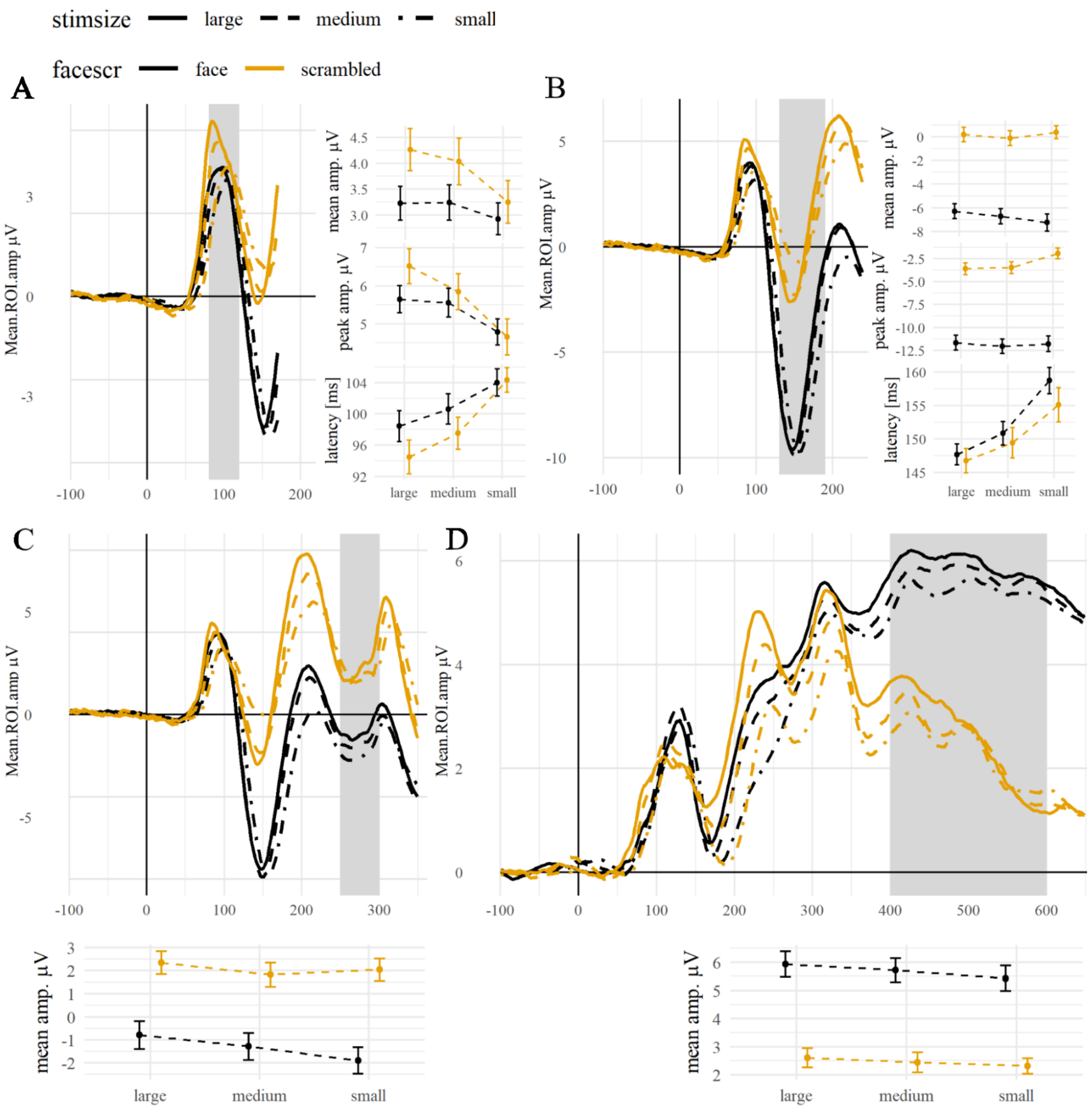

Notes: Grand average ERP time series of the averaged ROI channels. The highlighted areas display the respective ROI time window. Dot plots show the grand averages, contrasted for stimulus sizes and all emotion conditions. Error bars indicate +/- 1 SE of the mean. **A** left: P1 ERP time series, right: grand averages of the ROI mean and peak amplitudes and peak latencies. **B** left: N170 ERP time series, right: grand averages of the ROI mean and peak amplitudes and peak latencies. **C** top: EPN ERP time series, bottom: grand averages of the ROI mean amplitude. **D** top: LPC ERP time series, bottom: grand averages of the ROI mean amplitude.

Similar to P1 peak latencies, N170 peak latencies showed a trend for face intactness ($\chi^2(1) = 3.69$, $p = .055$), with longer latencies for intact faces ($\text{diff}_{\text{face-scrb}} = 1.36$). However, N170 peak latency was affected by stimulus size ($\chi^2(2) = 104.57$, $p < .001$), with shorter latencies for large stimuli, compared to medium and medium compared to small stimuli ($\text{diff}_{\text{L-M}} =$ -2.22, $p = .036$; $\text{diff}_{\text{M-S}} =$ -7.50, $p < .001$; $\text{diff}_{\text{L-S}} =$ -9.72, $p < .001$;). No interaction between face intactness and size was present ($\chi^2(2) = 0.53$, $p = .766$).

EPN: There was a difference between faces and scrambled stimuli for EPN amplitudes ($\chi^2(1) = 156.68$, $p < .001$), with more negative amplitudes for faces ($\text{diff}_{\text{face-scrb}} =$ -3.40). Stimulus size also modulated EPN amplitudes ($\chi^2(2) = 7.76$, $p = .021$) but only differed between large and small stimuli ($\text{diff}_{\text{L-S}} = 0.74$, $p = .022$) and there was no interaction between intactness and stimulus size ($\chi^2(2) = 2.52$, $p = .283$).

LPC: LPC amplitudes were only affected by intactness of the face ($\chi^2(1) = 184.64$, $p < .001$) with larger amplitudes for faces than for scrambled stimuli ($\text{diff}_{\text{face-scrb}} = 3.24$). Neither stimulus size ($\chi^2(2) = 2.94$, $p = .230$) nor the interaction between intactness and stimulus size were significant ($\chi^2(2) = 0.25$, $p = .881$).

We included scrambled versions of the faces as control stimuli to assess whether stimulus size would differentially affect stimuli of the same retinal size but different configuration. Throughout processing, ERPs differed between scrambled and intact faces. Early visual processing of intact faces averaged across emotion levels was differentially affected by stimulus size compared to scrambled stimuli, suggesting a distinct effect of size on faces, possibly due to their low-level properties. P1 mean and peak amplitudes were larger for scrambled stimuli than intact faces (see Gliga and Dehaene-Lambertz 2005; Schindler, Tirloni, et al. 2021 cf. Herrmann2004; Schindler, Bruchmann, et al. 2021; Zion-Golumbic and Bentin 2006). This difference was presumably driven by the reduced perceptual contrast of the (small) scrambled stimuli and a slight but systematic difference in luminance compared to the intact faces (see Figure S3), which may have contributed to increased P1 amplitudes (Johannes et al. 1995). Remarkably, P1 peak amplitudes showed an interaction between stimulus size and face intactness, where only medium and large scrambled stimuli differed from faces, but not small stimuli. A similar effect was found for peak latencies, with the shortest latency for large scrambled stimuli and the longest for small scrambled and intact stimuli. Although both low and high spatial frequencies have critical functions in face perception and have been shown to be used selectively depending on the task at hand (for reviews see Cesarei and Codispoti 2013; Ruiz-Soler and Beltran 2005), especially low frequencies have been suggested to affect coarse and fast initial visual processing (e.g. Bar et al. 2006; Willenbockel et al. 2012). In comparison, the random allocation of pixel chunks in scrambled stimuli particularly reduced low spatial frequencies but increased mid-spatial frequencies (see Figure S4). The low contrast of scrambled stimuli would cause them to blend more into the background (individual dark and light pixels would be perceived as gray), whereas edge contrasts are relatively well preserved even in small faces. Our results corroborate the well-established face sensitivity effect on the N170 (e.g., Bentin et al. 1996; Herrmann et al. 2004), with enhanced negative amplitudes for intact faces compared to the scrambled stimuli. However, when all emotion categories were collapsed, the size effect on the N170 for intact faces was not significant, suggesting that the N170 was not only affected by size but also by other low-level features (Mercure et al. 2008) that were specific to the emotional expressions. The EPN also differed between scrambled and intact stimuli, possibly because attention to scrambled faces might decrease dramatically once they are recognized as such, since no decision or response has been required. Stimulus size modulated the EPN for both intact and scrambled faces. Whether the (non-significant tendency for the) larger effect of size for intact faces additionally reflects attentional effects of the face or task-specific relevance would need to be further investigated in a study that matches the task-relevance of both types of stimuli. The impact of size on the LPC was not significant when face stimuli were collapsed and contrasted with scrambled stimuli. Considering the ERP time series, the size effect was most evident for all stimuli (before and) at the beginning of the LPC time window and decreased over time.

Preregistered statistical models

Table S1:
Statistical results for the P1 mean amplitudes by emotion and stimulus size

	β	SE	t	CI_l	CI_u	$Stab_{min}$	$Stab_{max}$	LRT:Model	χ^2	df	p	f^2
(Intercept)	3.13	0.30	10.32	2.57	3.71	3.03	3.23	(Intercept)	-	-	-	-
emotion_happy-g.m	-0.11	0.07	-1.56	-0.24	0.02	-0.13	-0.09	emotion	3.62	2	.163	0.01
emotion_angry-g.m	0.12	0.07	1.70	-0.02	0.26	0.11	0.13					
stimsize_medium-g.m	0.11	0.07	1.58	-0.03	0.26	0.08	0.15	stimsize	8.83	2	.012	0.03
stimsize_large-g.m	0.10	0.07	1.38	-0.04	0.23	0.06	0.14					
emotion_happy-g.m:stimsize_medium-g.m	-0.03	0.10	-0.35	-0.21	0.16	-0.06	-0.02	emotion:stimsize	2.50	4	.645	0.01
emotion_angry-g.m:stimsize_medium-g.m	0.07	0.10	0.71	-0.11	0.25	0.05	0.09					
emotion_happy-g.m:stimsize_large-g.m	0.12	0.10	1.18	-0.07	0.30	0.08	0.14					
emotion_angry-g.m:stimsize_large-g.m	-0.03	0.10	-0.28	-0.21	0.15	-0.04	-0.01					

Notes: β = model estimate, *SE* = standard error of the estimate, *CI* = lower and upper 95% bootstrapped confidence intervals, Stab = estimate ranges leaving out one participant at a time, LRT = Likelihood ratio test, f^2 = Cohen's f^2 effect size

Table S2:
Statistical results for the P1 peak amplitudes by emotion and stimulus size

	β	SE	t	CI_l	CI_u	$Stab_{min}$	$Stab_{max}$	LRT:Model	χ^2	df	p	f^2
(Intercept)	4.81	0.34	14.30	4.16	5.43	4.68	4.93	(Intercept)	-	-	-	-
emotion_happy-g.m	-0.13	0.08	-1.69	-0.27	0.03	-0.14	-0.11	emotion	4.14	2	.126	0.01
emotion_angry-g.m	0.14	0.08	1.80	0.00	0.28	0.12	0.15					
stimsize_medium-g.m	0.15	0.08	2.05	0.01	0.31	0.12	0.19	stimsize	42.68	2	<.001	0.14
stimsize_large-g.m	0.34	0.08	4.47	0.20	0.48	0.30	0.37					
emotion_happy-g.m:stimsize_medium-g.m	-0.05	0.11	-0.44	-0.26	0.18	-0.07	-0.03	emotion:stimsize	0.82	4	.935	0.00
emotion_angry-g.m:stimsize_medium-g.m	0.04	0.11	0.35	-0.18	0.24	0.02	0.07					
emotion_happy-g.m:stimsize_large-g.m	0.09	0.11	0.80	-0.13	0.29	0.06	0.10					
emotion_angry-g.m:stimsize_large-g.m	-0.02	0.11	-0.19	-0.22	0.19	-0.04	0.00					

Notes: β = model estimate, *SE* = standard error of the estimate, *CI* = lower and upper 95% bootstrapped confidence intervals, Stab = estimate ranges leaving out one participant at a time, LRT = Likelihood ratio test, f^2 = Cohen's f^2 effect size

Table S3:
Statistical results for the P1 peak latency by emotion and stimulus size

	β	SE	t	CI_l	CI_u	$Stab_{min}$	$Stab_{max}$	LRT:Model	χ^2	df	p	f^2
(Intercept)	100.51	1.64	61.24	97.23	103.90	100.07	100.94	(Intercept)	-	-	-	-
emotion_happy-g.m	-1.32	0.51	-2.61	-2.33	-0.38	-1.45	-1.09	emotion	6.94	2	.031	0.02
emotion_angry-g.m	0.76	0.51	1.50	-0.20	1.74	0.62	0.95					
stimsize_medium-g.m	-0.40	0.51	-0.78	-1.39	0.53	-0.60	-0.13	stimsize	40.24	2	<.001	0.13
stimsize_large-g.m	-2.63	0.51	-5.17	-3.58	-1.60	-2.85	-2.35					
emotion_happy-g.m:stimsize_medium-g.m	-0.40	0.72	-0.56	-1.82	0.94	-0.63	-0.19	emotion:stimsize	0.52	4	.971	0.00
emotion_angry-g.m:stimsize_medium-g.m	0.35	0.72	0.48	-1.05	1.73	0.10	0.56					
emotion_happy-g.m:stimsize_large-g.m	0.27	0.72	0.37	-1.05	1.69	-0.13	0.53					
emotion_angry-g.m:stimsize_large-g.m	-0.40	0.72	-0.56	-1.81	1.00	-0.63	-0.23					

Notes: β = model estimate, *SE* = standard error of the estimate, *CI* = lower and upper 95% bootstrapped confidence intervals, Stab = estimate ranges leaving out one participant at a time, LRT = Likelihood ratio test, f^2 = Cohen's f^2 effect sizeNote that the inspection of residuals indicated a potential misfit of the model, possibly due to the temporal boundary of the ROI time window.

Table S4:

Statistical results for the N170 mean amplitudes by emotion and stimulus size

	β	SE	t	CI_l	CI_u	$Stab_{min}$	$Stab_{max}$	LRT:Model	χ^2	df	p	f^2
(Intercept)	-6.75	0.65	-10.44	-7.98	-5.50	-6.90	-6.49	(Intercept)	-	-	-	-
emotion_happy-g.m	-0.06	0.09	-0.69	-0.24	0.10	-0.07	-0.04	emotion	19.78	2	<.001	0.06
emotion_angry-g.m	-0.30	0.09	-3.47	-0.47	-0.13	-0.32	-0.27					
stimsize_medium-g.m	0.03	0.09	0.33	-0.13	0.19	0.00	0.05	stimsize	40.72	2	<.001	0.14
stimsize_large-g.m	0.47	0.09	5.46	0.31	0.63	0.42	0.51					
emotion_happy-g.m:stimsize_medium-g.m	0.07	0.12	0.58	-0.17	0.30	0.04	0.09	emotion:stimsize	0.46	4	.977	0.00
emotion_angry-g.m:stimsize_medium-g.m	-0.01	0.12	-0.09	-0.24	0.23	-0.03	0.02					
emotion_happy-g.m:stimsize_large-g.m	-0.01	0.12	-0.10	-0.26	0.22	-0.03	0.01					
emotion_angry-g.m:stimsize_large-g.m	-0.01	0.12	-0.07	-0.25	0.23	-0.03	0.01					

Notes: β = model estimate, SE = standard error of the estimate, CI = lower and upper 95% bootstrapped confidence intervals, Stab = estimate ranges leaving out one participant at a time, LRT = Likelihood ratio test, f^2 = Cohen's f^2 effect size

Table S5:

Statistical results for the N170 peak amplitudes by emotion and stimulus size

	β	SE	t	CI_l	CI_u	$Stab_{min}$	$Stab_{max}$	LRT:Model	χ^2	df	p	f^2
(Intercept)	-11.13	0.80	-13.90	-12.73	-9.51	-11.31	-10.78	(Intercept)	-	-	-	-
emotion_happy-g.m	-0.08	0.07	-1.16	-0.22	0.06	-0.10	-0.06	emotion	30.63	2	<.001	0.10
emotion_angry-g.m	-0.29	0.07	-4.16	-0.44	-0.16	-0.32	-0.27					
stimsize_medium-g.m	-0.24	0.07	-3.36	-0.37	-0.11	-0.26	-0.22	stimsize	11.36	2	.003	0.04
stimsize_large-g.m	0.11	0.07	1.57	-0.03	0.25	0.07	0.16					
emotion_happy-g.m:stimsize_medium-g.m	0.06	0.10	0.63	-0.13	0.27	0.04	0.09	emotion:stimsize	0.93	4	.920	0.00
emotion_angry-g.m:stimsize_medium-g.m	-0.01	0.10	-0.15	-0.19	0.18	-0.04	0.02					
emotion_happy-g.m:stimsize_large-g.m	-0.02	0.10	-0.22	-0.21	0.17	-0.05	0.00					
emotion_angry-g.m:stimsize_large-g.m	0.05	0.10	0.54	-0.13	0.25	0.03	0.08					

Notes: β = model estimate, SE = standard error of the estimate, CI = lower and upper 95% bootstrapped confidence intervals, Stab = estimate ranges leaving out one participant at a time, LRT = Likelihood ratio test, f^2 = Cohen's f^2 effect size

Table S6:

Statistical results for the N170 peak latency by emotion and stimulus size

	β	SE	t	CI_l	CI_u	$Stab_{min}$	$Stab_{max}$	LRT:Model	χ^2	df	p	f^2
(Intercept)	152.28	1.76	86.57	148.60	155.69	151.59	152.71	(Intercept)	-	-	-	-
emotion_happy-g.m	0.05	0.26	0.18	-0.45	0.57	-0.06	0.14	emotion	3.66	2	.160	0.01
emotion_angry-g.m	0.41	0.26	1.54	-0.07	0.94	0.32	0.50					
stimsize_medium-g.m	-1.90	0.26	-7.21	-2.42	-1.38	-1.98	-1.76	stimsize	337.03	2	<.001	1.87
stimsize_large-g.m	-4.31	0.26	-16.34	-4.78	-3.76	-4.45	-4.12					
emotion_happy-g.m:stimsize_medium-g.m	0.02	0.37	0.04	-0.78	0.76	-0.08	0.11	emotion:stimsize	2.21	4	.697	0.01
emotion_angry-g.m:stimsize_medium-g.m	0.29	0.37	0.78	-0.45	1.03	0.17	0.39					
emotion_happy-g.m:stimsize_large-g.m	-0.31	0.37	-0.83	-1.01	0.43	-0.37	-0.21					
emotion_angry-g.m:stimsize_large-g.m	-0.18	0.37	-0.48	-0.92	0.56	-0.29	-0.07					

Notes: β = model estimate, SE = standard error of the estimate, CI = lower and upper 95% bootstrapped confidence intervals, Stab = estimate ranges leaving out one participant at a time, LRT = Likelihood ratio test, f^2 = Cohen's f^2 effect size

Table S7:

Statistical results for the EPN mean amplitudes by emotion and stimulus size

	β	SE	t	CI_l	CI_u	$Stab_{min}$	$Stab_{max}$	LRT:Model	χ^2	df	p	f^2
(Intercept)	-1.28	0.57	-2.23	-2.38	-0.09	-1.56	-1.10	(Intercept)	-	-	-	-
emotion_happy-g.m	-0.28	0.08	-3.64	-0.43	-0.11	-0.30	-0.25	emotion	29.42	2	<.001	0.10
emotion_angry-g.m	-0.13	0.08	-1.73	-0.27	0.02	-0.16	-0.11					
stimsize_medium-g.m	0.03	0.08	0.44	-0.12	0.17	0.01	0.06	stimsize	64.14	2	<.001	0.22
stimsize_large-g.m	0.54	0.08	6.98	0.38	0.69	0.49	0.59					
emotion_happy-g.m:stimsize_medium-g.m	0.06	0.11	0.58	-0.15	0.27	0.04	0.08	emotion:stimsize	5.00	4	.287	0.02
emotion_angry-g.m:stimsize_medium-g.m	0.09	0.11	0.85	-0.11	0.30	0.06	0.11					
emotion_happy-g.m:stimsize_large-g.m	0.11	0.11	1.05	-0.11	0.33	0.09	0.14					
emotion_angry-g.m:stimsize_large-g.m	-0.06	0.11	-0.58	-0.28	0.17	-0.08	-0.03					

Notes: β = model estimate, *SE* = standard error of the estimate, *CI* = lower and upper 95% bootstrapped confidence intervals, Stab = estimate ranges leaving out one participant at a time, LRT = Likelihood ratio test, f^2 = Cohen's f^2 effect size

Table S8:

Statistical results for the LPC mean amplitudes by emotion and stimulus size

	β	SE	t	CI_l	CI_u	$Stab_{min}$	$Stab_{max}$	LRT:Model	χ^2	df	p	f^2
(Intercept)	5.69	0.44	13.02	4.85	6.51	5.54	5.82	(Intercept)	-	-	-	-
emotion_happy-g.m	0.24	0.06	4.08	0.13	0.36	0.22	0.27	emotion	17.32	2	<.001	0.06
emotion_angry-g.m	-0.08	0.06	-1.30	-0.20	0.04	-0.10	-0.05					
stimsize_medium-g.m	0.02	0.06	0.41	-0.09	0.14	0.01	0.05	stimsize	23.90	2	<.001	0.08
stimsize_large-g.m	0.24	0.06	4.04	0.12	0.36	0.20	0.25					
emotion_happy-g.m:stimsize_medium-g.m	0.10	0.08	1.23	-0.06	0.26	0.07	0.13	emotion:stimsize	2.20	4	.700	0.01
emotion_angry-g.m:stimsize_medium-g.m	-0.10	0.08	-1.18	-0.26	0.07	-0.13	-0.05					
emotion_happy-g.m:stimsize_large-g.m	-0.07	0.08	-0.90	-0.24	0.08	-0.10	-0.04					
emotion_angry-g.m:stimsize_large-g.m	0.08	0.08	0.97	-0.07	0.25	0.04	0.11					

Notes: β = model estimate, *SE* = standard error of the estimate, *CI* = lower and upper 95% bootstrapped confidence intervals, Stab = estimate ranges leaving out one participant at a time, LRT = Likelihood ratio test, f^2 = Cohen's f^2 effect size

Table S9:

Statistical results for accuracy in the naturalness classification task by emotion and stimulus size

	β	SE	z	CI_l	CI_u	$Stab_{min}$	$Stab_{max}$	LRT:Model	χ^2	df	p
(Intercept)	0.14	0.06	2.36	0.02	0.26	0.11	0.15	(Intercept)	-	-	-
emotion_happy-g.m	0.32	0.05	6.03	0.22	0.43	0.30	0.34	emotion	26.90	2	<.001
emotion_angry-g.m	-0.01	0.03	-0.26	-0.07	0.06	-0.02	0.00				
stimsize_large-g.m	0.01	0.02	0.36	-0.04	0.06	0.00	0.02	stimsize	0.13	2	.938
stimsize_medium-g.m	0.00	0.03	-0.07	-0.06	0.06	-0.01	0.01				
emotion_happy-g.m:stimsize_large-g.m	0.06	0.03	2.42	0.02	0.12	0.04	0.08	emotion:stimsize	16.17	4	.003
emotion_angry-g.m:stimsize_large-g.m	0.03	0.03	1.19	-0.02	0.08	0.02	0.05				
emotion_happy-g.m:stimsize_medium-g.m	-0.03	0.03	-1.15	-0.08	0.02	-0.05	-0.01				
emotion_angry-g.m:stimsize_medium-g.m	0.01	0.03	0.53	-0.04	0.07	0.00	0.04				

Notes: beta = model estimate, SE = standard error of the estimate, CI = lower and upper 95
Stab = estimate ranges leaving out one participant at a time, LRT = Likelihood ratio test

Table S10:

Statistical results for mean response times by emotion and stimulus size

	β	SE	t	CI_l	CI_u	$Stab_{min}$	$Stab_{max}$	LRT:Model	χ^2	df	p	f^2
(Intercept)	1041.36	43.43	23.98	958.91	1121.84	1023.94	1053.68	(Intercept)	-	-	-	-
emotion_happy-g.m	-39.22	5.25	-7.47	-49.76	-28.80	-41.26	-35.89	emotion	85.57	2	<.001	0.31
emotion_angry-g.m	48.30	5.25	9.20	38.11	59.20	44.82	49.86					
stimsize_large-g.m	13.31	5.25	2.54	3.52	23.72	11.05	15.01	stimsize	7.37	2	.025	0.02
stimsize_medium-g.m	-10.83	5.25	-2.06	-20.97	-1.10	-13.21	-8.41					
emotion_happy-g.m:stimsize_large-g.m	-0.92	7.42	-0.12	-15.58	12.97	-3.45	2.49	emotion:stimsize	5.25	4	.263	0.02
emotion_angry-g.m:stimsize_large-g.m	-5.87	7.42	-0.79	-20.48	9.30	-8.04	-2.89					
emotion_happy-g.m:stimsize_medium-g.m	11.36	7.42	1.53	-3.15	25.56	9.31	13.61					
emotion_angry-g.m:stimsize_medium-g.m	-8.86	7.42	-1.19	-23.50	5.07	-10.62	-4.01					

Notes: β = model estimate, SE = standard error of the estimate, CI = lower and upper 95% bootstrapped confidence intervals, Stab = estimate ranges leaving out one participant at a time, LRT = Likelihood ratio test, f^2 = Cohen's f^2 effect size

Table S11:

Statistical results for the P1 mean amplitudes by facial intactness and stimulus size

	β	SE	t	CI_l	CI_u	$Stab_{min}$	$Stab_{max}$	LRT:Model	χ^2	df	p	f^2
(Intercept)	3.49	0.34	10.36	2.80	4.22	3.34	3.60	(Intercept)	-	-	-	-
facescr_face-g.m	-0.36	0.08	-4.69	-0.51	-0.21	-0.38	-0.32	facescr	21.42	1	<.001	0.11
stimsize_medium-g.m	0.15	0.11	1.36	-0.06	0.36	0.11	0.19	stimsize	14.03	2	<.001	0.07
stimsize_large-g.m	0.26	0.11	2.36	0.03	0.46	0.22	0.31					
facescr_face-g.m:stimsize_medium-g.m	-0.04	0.11	-0.34	-0.24	0.16	-0.05	-0.02	facescr:stimsize	3.80	2	.149	0.02
facescr_face-g.m:stimsize_large-g.m	-0.16	0.11	-1.48	-0.37	0.06	-0.18	-0.15					

Notes: β = model estimate, SE = standard error of the estimate, CI = lower and upper 95% bootstrapped confidence intervals, Stab = estimate ranges leaving out one participant at a time, LRT = Likelihood ratio test, f^2 = Cohen's f^2 effect size

Table S12:

Statistical results for the P1 peak amplitudes by facial intactness and stimulus size

	β	SE	t	CI_l	CI_u	$Stab_{min}$	$Stab_{max}$	LRT:Model	χ^2	df	p	f^2
(Intercept)	5.24	0.38	13.88	4.49	5.97	5.08	5.37	(Intercept)	-	-	-	-
facescr_face-g.m	-0.43	0.08	-5.37	-0.60	-0.27	-0.47	-0.40	facescr	27.55	1	<.001	0.15
stimsize_medium-g.m	0.16	0.11	1.43	-0.06	0.38	0.13	0.19	stimsize	44.34	2	<.001	0.25
stimsize_large-g.m	0.59	0.11	5.18	0.37	0.82	0.54	0.63					
facescr_face-g.m:stimsize_medium-g.m	-0.01	0.11	-0.08	-0.23	0.21	-0.02	0.01	facescr:stimsize	6.84	2	.033	0.03
facescr_face-g.m:stimsize_large-g.m	-0.25	0.11	-2.22	-0.47	-0.02	-0.28	-0.23					

Notes: β = model estimate, SE = standard error of the estimate, CI = lower and upper 95% bootstrapped confidence intervals, Stab = estimate ranges leaving out one participant at a time, LRT = Likelihood ratio test, f^2 = Cohen's f^2 effect size

Table S13:

Statistical results for the P1 peak latency by facial intactness and stimulus size

	β	SE	t	CI_l	CI_u	$Stab_{min}$	$Stab_{max}$	LRT:Model	χ^2	df	p	f^2
(Intercept)	99.64	1.48	67.29	96.49	102.57	99.17	100.06	(Intercept)	-	-	-	-
facescr_face-g.m	0.87	0.51	1.69	-0.10	1.87	0.55	1.19	facescr	2.92	1	.087	0.01
stimsize_medium-g.m	-0.83	0.72	-1.15	-2.27	0.64	-1.17	-0.58	stimsize	37.11	2	<.001	0.20
stimsize_large-g.m	-3.46	0.72	-4.79	-4.87	-2.06	-3.80	-3.25					
facescr_face-g.m:stimsize_medium-g.m	0.44	0.72	0.60	-0.99	2.01	0.19	0.62	facescr:stimsize	3.26	2	.196	0.02
facescr_face-g.m:stimsize_large-g.m	0.84	0.72	1.16	-0.56	2.20	0.60	1.04					

Notes: β = model estimate, SE = standard error of the estimate, CI = lower and upper 95% bootstrapped confidence intervals, Stab = estimate ranges leaving out one participant at a time, LRT = Likelihood ratio test, f^2 = Cohen's f^2 effect sizeNote that the inspection of residuals indicated a potential misfit of the model, possibly due to the temporal boundary of the ROI timewindow.

Table S14:

Statistical results for the N170 mean amplitudes by facial intactness and stimulus size

	β	SE	t	CI_l	CI_u	$Stab_{min}$	$Stab_{max}$	LRT:Model	χ^2	df	p	f^2
(Intercept)	-3.30	0.56	-5.92	-4.41	-2.26	-3.48	-3.00	(Intercept)	-	-	-	-
facescr_face-g.m	-3.45	0.13	-26.29	-3.69	-3.19	-3.54	-3.34	facescr	302.79	1	<.001	3.54
stimsize_medium-g.m	-0.12	0.19	-0.65	-0.49	0.24	-0.14	-0.10	stimsize	1.86	2	.395	0.01
stimsize_large-g.m	0.25	0.19	1.35	-0.13	0.61	0.22	0.30					
facescr_face-g.m:stimsize_medium-g.m	0.15	0.19	0.80	-0.22	0.51	0.13	0.17	facescr:stimsize	3.93	2	.140	0.02
facescr_face-g.m:stimsize_large-g.m	0.22	0.19	1.16	-0.15	0.58	0.18	0.23					

Notes: β = model estimate, *SE* = standard error of the estimate, *CI* = lower and upper 95% bootstrapped confidence intervals, Stab = estimate ranges leaving out one participant at a time, LRT = Likelihood ratio test, f^2 = Cohen's f^2 effect size

Table S15:

Statistical results for the N170 peak amplitudes by facial intactness and stimulus size

	β	SE	t	CI_l	CI_u	$Stab_{min}$	$Stab_{max}$	LRT:Model	χ^2	df	p	f^2
(Intercept)	-7.06	0.64	-11.02	-8.34	-5.84	-7.23	-6.73	(Intercept)	-	-	-	-
facescr_face-g.m	-4.07	0.15	-26.97	-4.37	-3.75	-4.17	-3.95	facescr	310.77	1	<.001	3.73
stimsize_medium-g.m	-0.35	0.21	-1.66	-0.76	0.07	-0.38	-0.33	stimsize	8.01	2	.018	0.04
stimsize_large-g.m	-0.24	0.21	-1.15	-0.63	0.17	-0.28	-0.20					
facescr_face-g.m:stimsize_medium-g.m	0.12	0.21	0.55	-0.28	0.49	0.09	0.14	facescr:stimsize	5.38	2	.068	0.03
facescr_face-g.m:stimsize_large-g.m	0.36	0.21	1.67	-0.11	0.76	0.32	0.38					

Notes: β = model estimate, *SE* = standard error of the estimate, *CI* = lower and upper 95% bootstrapped confidence intervals, Stab = estimate ranges leaving out one participant at a time, LRT = Likelihood ratio test, f^2 = Cohen's f^2 effect size

Table S16:

Statistical results for the N170 peak latency by facial intactness and stimulus size

	β	SE	t	CI_l	CI_u	$Stab_{min}$	$Stab_{max}$	LRT:Model	χ^2	df	p	f^2
(Intercept)	150.96	1.81	83.52	147.30	154.58	150.12	151.34	(Intercept)	-	-	-	-
facescr_face-g.m	0.68	0.36	1.90	0.00	1.41	0.54	0.91	facescr	3.69	1	.055	0.02
stimsize_medium-g.m	-1.76	0.51	-3.47	-2.79	-0.73	-1.89	-1.66	stimsize	104.57	2	<.001	0.71
stimsize_large-g.m	-3.98	0.51	-7.86	-4.95	-2.93	-4.15	-3.85					
facescr_face-g.m:stimsize_medium-g.m	-0.22	0.51	-0.44	-1.21	0.75	-0.30	-0.11	facescr:stimsize	0.53	2	.766	0.00
facescr_face-g.m:stimsize_large-g.m	-0.14	0.51	-0.27	-1.10	0.87	-0.25	0.01					

Notes: β = model estimate, *SE* = standard error of the estimate, *CI* = lower and upper 95% bootstrapped confidence intervals, Stab = estimate ranges leaving out one participant at a time, LRT = Likelihood ratio test, f^2 = Cohen's f^2 effect sizeNote that one subject was removed from this model due to influential observations.

Table S17:

Statistical results for the EPN mean amplitudes by facial intactness and stimulus size

	β	SE	t	CI_l	CI_u	$Stab_{min}$	$Stab_{max}$	LRT:Model	χ^2	df	p	f^2
(Intercept)	0.42	0.48	0.88	-0.47	1.34	0.17	0.59	(Intercept)	-	-	-	-
facescr_face-g.m	-1.70	0.11	-15.23	-1.92	-1.48	-1.75	-1.58	facescr	156.68	1	<.001	1.19
stimsize_medium-g.m	-0.10	0.16	-0.62	-0.41	0.22	-0.12	-0.08	stimsize	7.76	2	.021	0.04
stimsize_large-g.m	0.42	0.16	2.66	0.09	0.72	0.38	0.44					
facescr_face-g.m:stimsize_medium-g.m	0.13	0.16	0.84	-0.21	0.44	0.11	0.15	facescr:stimsize	2.52	2	.283	0.01
facescr_face-g.m:stimsize_large-g.m	0.12	0.16	0.73	-0.18	0.42	0.09	0.15					

Notes: β = model estimate, *SE* = standard error of the estimate, *CI* = lower and upper 95% bootstrapped confidence intervals, Stab = estimate ranges leaving out one participant at a time, LRT = Likelihood ratio test, f^2 = Cohen's f^2 effect size

Table S18:

Statistical results for the LPC mean amplitudes by facial intactness and stimulus size

	β	SE	t	CI_l	CI_u	$Stab_{min}$	$Stab_{max}$	LRT:Model	χ^2	df	p	f^2
(Intercept)	4.07	0.33	12.46	3.44	4.68	3.96	4.17	(Intercept)	-	-	-	-
facescr_face-g.m	1.62	0.09	17.20	1.43	1.81	1.54	1.69	facescr	184.64	1	<.001	1.52
stimsize_medium-g.m	0.00	0.13	0.03	-0.26	0.27	-0.02	0.02	stimsize	2.94	2	.230	0.01
stimsize_large-g.m	0.19	0.13	1.46	-0.06	0.45	0.16	0.21					
facescr_face-g.m:stimsize_medium-g.m	0.02	0.13	0.15	-0.22	0.28	0.01	0.04	facescr:stimsize	0.25	2	.881	0.00
facescr_face-g.m:stimsize_large-g.m	0.04	0.13	0.34	-0.21	0.29	0.03	0.06					

Notes: β = model estimate, *SE* = standard error of the estimate, *CI* = lower and upper 95% bootstrapped confidence intervals, Stab = estimate ranges leaving out one participant at a time, LRT = Likelihood ratio test, f^2 = Cohen's f^2 effect size

Table S19:

Statistical results for the P1 mean amplitudes by emotion (incl. scrambled) and stimulus size

	β	SE	t	CI_l	CI_u	$Stab_{min}$	$Stab_{max}$	LRT:Model	χ^2	df	p	f^2
(Intercept)	3.31	0.32	10.43	2.68	3.91	3.19	3.42	(Intercept)	-	-	-	-
emotion_scrambled-g.m	0.54	0.09	6.19	0.36	0.72	0.47	0.58	emotion	40.07	3	<.001	0.10
emotion_happy-g.m	-0.29	0.09	-3.31	-0.45	-0.13	-0.31	-0.26					
emotion_angry-g.m	-0.06	0.09	-0.70	-0.23	0.11	-0.08	-0.04					
stimsize_medium-g.m	0.13	0.07	1.80	-0.01	0.27	0.09	0.17	stimsize	18.52	2	<.001	0.04
stimsize_large-g.m	0.18	0.07	2.47	0.03	0.32	0.14	0.22					
emotion_scrambled-g.m:stimsize_medium-g.m	0.06	0.12	0.45	-0.19	0.28	0.04	0.08	emotion:stimsize	8.40	6	.210	0.02
emotion_happy-g.m:stimsize_medium-g.m	-0.05	0.12	-0.43	-0.28	0.17	-0.07	-0.04					
emotion_angry-g.m:stimsize_medium-g.m	0.05	0.12	0.42	-0.20	0.28	0.03	0.07					
emotion_scrambled-g.m:stimsize_large-g.m	0.24	0.12	1.95	-0.02	0.48	0.22	0.27					
emotion_happy-g.m:stimsize_large-g.m	0.04	0.12	0.30	-0.20	0.28	0.00	0.05					
emotion_angry-g.m:stimsize_large-g.m	-0.11	0.12	-0.88	-0.35	0.12	-0.13	-0.08					

Notes: β = model estimate, *SE* = standard error of the estimate, *CI* = lower and upper 95% bootstrapped confidence intervals, Stab = estimate ranges leaving out one participant at a time, LRT = Likelihood ratio test, f^2 = Cohen's f^2 effect size

Table S20:

Statistical results for the P1 peak amplitudes by emotion (incl. scrambled) and stimulus size

	β	SE	t	CI_l	CI_u	$Stab_{min}$	$Stab_{max}$	LRT:Model	χ^2	df	p	f^2
(Intercept)	5.02	0.35	14.18	4.34	5.73	4.88	5.15	(Intercept)	-	-	-	-
emotion_scrambled-g.m	0.65	0.09	6.99	0.48	0.83	0.60	0.70	emotion	50.20	3	<.001	0.12
emotion_happy-g.m	-0.34	0.09	-3.70	-0.52	-0.15	-0.36	-0.32					
emotion_angry-g.m	-0.08	0.09	-0.87	-0.26	0.10	-0.10	-0.06					
stimsize_medium-g.m	0.16	0.08	2.10	0.01	0.30	0.13	0.19	stimsize	69.08	2	<.001	0.17
stimsize_large-g.m	0.46	0.08	6.12	0.31	0.63	0.42	0.50					
emotion_scrambled-g.m:stimsize_medium-g.m	0.01	0.13	0.10	-0.23	0.27	-0.01	0.04	emotion:stimsize	12.25	6	.057	0.03
emotion_happy-g.m:stimsize_medium-g.m	-0.05	0.13	-0.39	-0.30	0.21	-0.08	-0.03					
emotion_angry-g.m:stimsize_medium-g.m	0.03	0.13	0.25	-0.23	0.27	0.01	0.07					
emotion_scrambled-g.m:stimsize_large-g.m	0.38	0.13	2.89	0.11	0.64	0.35	0.42					
emotion_happy-g.m:stimsize_large-g.m	-0.04	0.13	-0.31	-0.30	0.22	-0.07	-0.02					
emotion_angry-g.m:stimsize_large-g.m	-0.15	0.13	-1.12	-0.39	0.11	-0.17	-0.12					

Notes: β = model estimate, *SE* = standard error of the estimate, *CI* = lower and upper 95% bootstrapped confidence intervals, Stab = estimate ranges leaving out one participant at a time, LRT = Likelihood ratio test, f^2 = Cohen's f^2 effect size

Table S21:

Statistical results for the P1 peak latency by emotion (incl. scrambled) and stimulus size

	β	SE	t	CI_l	CI_u	$Stab_{min}$	$Stab_{max}$	LRT:Model	χ^2	df	p	f^2
(Intercept)	100.08	1.53	65.57	96.86	102.92	99.62	100.50	(Intercept)	-	-	-	-
emotion_happy-g.m	-0.89	0.62	-1.43	-2.12	0.32	-1.05	-0.75	emotion	9.64	3	.022	0.02
emotion_angry-g.m	1.19	0.62	1.92	0.00	2.44	0.97	1.54					
emotion_neutral-g.m	1.00	0.62	1.60	-0.20	2.23	0.79	1.14					
stimsize_medium-g.m	-0.61	0.51	-1.21	-1.60	0.35	-0.88	-0.38	stimsize	57.24	2	<.001	0.14
stimsize_large-g.m	-3.04	0.51	-6.00	-3.99	-2.04	-3.28	-2.80					
emotion_happy-g.m:stimsize_medium-g.m	-0.18	0.88	-0.21	-1.94	1.45	-0.36	0.06	emotion:stimsize	5.35	6	.499	0.01
emotion_angry-g.m:stimsize_medium-g.m	0.57	0.88	0.64	-1.27	2.22	0.31	0.76					
emotion_neutral-g.m:stimsize_medium-g.m	0.27	0.88	0.31	-1.42	2.06	-0.19	0.61					
emotion_happy-g.m:stimsize_large-g.m	0.68	0.88	0.78	-1.10	2.49	0.29	0.93					
emotion_angry-g.m:stimsize_large-g.m	0.02	0.88	0.02	-1.68	1.75	-0.17	0.21					
emotion_neutral-g.m:stimsize_large-g.m	0.55	0.88	0.63	-1.16	2.30	0.38	0.84					

Notes: β = model estimate, *SE* = standard error of the estimate, *CI* = lower and upper 95% bootstrapped confidence intervals, Stab = estimate ranges leaving out one participant at a time, LRT = Likelihood ratio test, f^2 = Cohen's f^2 effect size

Table S22:

Statistical results for the N170 mean amplitudes by emotion (incl. scrambled) and stimulus size

	β	SE	t	CI_l	CI_u	$Stab_{min}$	$Stab_{max}$	LRT:Model	χ^2	df	p	f^2
(Intercept)	-5.03	0.59	-8.52	-6.14	-3.86	-5.19	-4.74	(Intercept)	-	-	-	-
emotion_scrambled-g.m	5.17	0.14	35.84	4.89	5.45	5.01	5.31	emotion	611.31	3	<.001	3.01
emotion_happy-g.m	-1.78	0.14	-12.35	-2.07	-1.48	-1.84	-1.73					
emotion_angry-g.m	-2.02	0.14	-13.99	-2.30	-1.75	-2.07	-1.96					
stimsize_medium-g.m	-0.05	0.12	-0.39	-0.29	0.19	-0.07	-0.03	stimsize	11.06	2	.004	0.03
stimsize_large-g.m	0.36	0.12	3.04	0.14	0.58	0.32	0.40					
emotion_scrambled-g.m:stimsize_medium-g.m	-0.22	0.20	-1.09	-0.63	0.16	-0.26	-0.20	emotion:stimsize	7.50	6	.277	0.02
emotion_happy-g.m:stimsize_medium-g.m	0.14	0.20	0.71	-0.22	0.56	0.12	0.17					
emotion_angry-g.m:stimsize_medium-g.m	0.06	0.20	0.31	-0.35	0.44	0.04	0.10					
emotion_scrambled-g.m:stimsize_large-g.m	-0.32	0.20	-1.58	-0.71	0.06	-0.35	-0.27					
emotion_happy-g.m:stimsize_large-g.m	0.10	0.20	0.47	-0.31	0.47	0.07	0.12					
emotion_angry-g.m:stimsize_large-g.m	0.10	0.20	0.49	-0.29	0.50	0.07	0.13					

Notes: β = model estimate, *SE* = standard error of the estimate, *CI* = lower and upper 95% bootstrapped confidence intervals, Stab = estimate ranges leaving out one participant at a time, LRT = Likelihood ratio test, f^2 = Cohen's f^2 effect size

Table S23:

Statistical results for the N170 peak amplitudes by emotion (incl. scrambled) and stimulus size

	β	SE	t	CI_l	CI_u	$Stab_{min}$	$Stab_{max}$	LRT:Model	χ^2	df	p	f^2
(Intercept)	-9.09	0.71	-12.85	-10.44	-7.75	-9.27	-8.78	(Intercept)	-	-	-	-
emotion_scrambled-g.m	6.10	0.16	38.21	5.81	6.42	5.92	6.25	emotion	653.86	3	<.001	3.42
emotion_happy-g.m	-2.11	0.16	-13.25	-2.43	-1.78	-2.18	-2.05					
emotion_angry-g.m	-2.33	0.16	-14.58	-2.65	-2.02	-2.39	-2.27					
stimsize_medium-g.m	-0.30	0.13	-2.27	-0.55	-0.04	-0.31	-0.28	stimsize	8.90	2	.012	0.02
stimsize_large-g.m	-0.07	0.13	-0.51	-0.32	0.18	-0.10	-0.03					
emotion_scrambled-g.m:stimsize_medium-g.m	-0.17	0.23	-0.77	-0.60	0.27	-0.21	-0.14	emotion:stimsize	11.02	6	.088	0.03
emotion_happy-g.m:stimsize_medium-g.m	0.12	0.23	0.54	-0.31	0.55	0.09	0.15					
emotion_angry-g.m:stimsize_medium-g.m	0.04	0.23	0.19	-0.37	0.49	0.01	0.08					
emotion_scrambled-g.m:stimsize_large-g.m	-0.53	0.23	-2.36	-0.95	-0.08	-0.56	-0.48					
emotion_happy-g.m:stimsize_large-g.m	0.16	0.23	0.69	-0.30	0.60	0.12	0.19					
emotion_angry-g.m:stimsize_large-g.m	0.23	0.23	1.03	-0.19	0.65	0.19	0.27					

Notes: β = model estimate, *SE* = standard error of the estimate, *CI* = lower and upper 95% bootstrapped confidence intervals, Stab = estimate ranges leaving out one participant at a time, LRT = Likelihood ratio test, f^2 = Cohen's f^2 effect size

Table S24:

Statistical results for the N170 peak latency by emotion (incl. scrambled) and stimulus size

	β	SE	t	CI_l	CI_u	$Stab_{min}$	$Stab_{max}$	LRT:Model	χ^2	df	p	f^2
(Intercept)	151.82	1.76	86.35	148.27	155.19	151.11	152.22	(Intercept)	-	-	-	-
emotion_happy-g.m	0.50	0.43	1.18	-0.38	1.34	0.38	0.69	emotion	11.83	3	.008	0.03
emotion_angry-g.m	0.86	0.43	2.01	0.04	1.70	0.77	1.00					
emotion_neutral-g.m	0.00	0.43	0.00	-0.83	0.79	-0.17	0.21					
stimsize_medium-g.m	-1.68	0.35	-4.79	-2.35	-0.98	-1.87	-1.56	stimsize	229.54	2	<.001	0.68
stimsize_large-g.m	-4.15	0.35	-11.86	-4.85	-3.51	-4.26	-4.05					
emotion_happy-g.m:stimsize_medium-g.m	-0.21	0.61	-0.35	-1.42	1.00	-0.33	-0.08	emotion:stimsize	4.71	6	.582	0.01
emotion_angry-g.m:stimsize_medium-g.m	0.07	0.61	0.11	-1.18	1.21	-0.08	0.17					
emotion_neutral-g.m:stimsize_medium-g.m	-0.54	0.61	-0.89	-1.73	0.67	-0.61	-0.38					
emotion_happy-g.m:stimsize_large-g.m	-0.47	0.61	-0.77	-1.68	0.78	-0.56	-0.34					
emotion_angry-g.m:stimsize_large-g.m	-0.34	0.61	-0.56	-1.62	0.84	-0.43	-0.16					
emotion_neutral-g.m:stimsize_large-g.m	0.33	0.61	0.54	-0.88	1.47	0.16	0.40					

Notes: β = model estimate, *SE* = standard error of the estimate, *CI* = lower and upper 95% bootstrapped confidence intervals, Stab = estimate ranges leaving out one participant at a time, LRT = Likelihood ratio test, f^2 = Cohen's f^2 effect sizeNote that one subject was removed from this model due to influential observations.

Table S25:

Statistical results for the EPN mean amplitudes by emotion (incl. scrambled) and stimulus size

	β	SE	t	CI_l	CI_u	$Stab_{min}$	$Stab_{max}$	LRT:Model	χ^2	df	p	f^2
(Intercept)	-0.43	0.52	-0.82	-1.40	0.62	-0.69	-0.26	(Intercept)	-	-	-	-
emotion_scrambled-g.m	2.55	0.12	20.46	2.28	2.82	2.37	2.63	emotion	306.20	3	<.001	1.01
emotion_happy-g.m	-1.13	0.12	-9.06	-1.37	-0.87	-1.16	-1.05					
emotion_angry-g.m	-0.98	0.12	-7.89	-1.24	-0.75	-1.03	-0.91					
stimsize_medium-g.m	-0.03	0.10	-0.32	-0.22	0.17	-0.05	-0.01	stimsize	27.33	2	<.001	0.06
stimsize_large-g.m	0.48	0.10	4.69	0.26	0.67	0.44	0.51					
emotion_scrambled-g.m:stimsize_medium-g.m	-0.20	0.18	-1.12	-0.57	0.16	-0.23	-0.16	emotion:stimsize	6.68	6	.351	0.02
emotion_happy-g.m:stimsize_medium-g.m	0.13	0.18	0.73	-0.22	0.45	0.10	0.15					
emotion_angry-g.m:stimsize_medium-g.m	0.16	0.18	0.90	-0.20	0.51	0.13	0.18					
emotion_scrambled-g.m:stimsize_large-g.m	-0.17	0.18	-0.99	-0.50	0.16	-0.22	-0.13					
emotion_happy-g.m:stimsize_large-g.m	0.17	0.18	0.98	-0.15	0.52	0.15	0.20					
emotion_angry-g.m:stimsize_large-g.m	-0.01	0.18	-0.03	-0.35	0.34	-0.04	0.03					

Notes: β = model estimate, *SE* = standard error of the estimate, *CI* = lower and upper 95% bootstrapped confidence intervals, Stab = estimate ranges leaving out one participant at a time, LRT = Likelihood ratio test, f^2 = Cohen's f^2 effect size

Table S26:

Statistical results for the LPC mean amplitudes by emotion (incl. scrambled) and stimulus size

	β	SE	t	CI_l	CI_u	$Stab_{min}$	$Stab_{max}$	LRT:Model	χ^2	df	p	f^2
(Intercept)	4.88	0.37	13.08	4.24	5.59	4.75	4.99	(Intercept)	-	-	-	-
emotion_scrambled-g.m	-2.43	0.11	-22.92	-2.62	-2.22	-2.53	-2.31	emotion	354.61	3	<.001	1.24
emotion_happy-g.m	1.05	0.11	9.91	0.85	1.25	1.01	1.09					
emotion_angry-g.m	0.73	0.11	6.92	0.53	0.95	0.69	0.78					
stimsize_medium-g.m	0.01	0.09	0.17	-0.15	0.19	0.00	0.03	stimsize	9.04	2	.011	0.02
stimsize_large-g.m	0.22	0.09	2.50	0.05	0.38	0.19	0.23					
emotion_scrambled-g.m:stimsize_medium-g.m	-0.03	0.15	-0.20	-0.33	0.26	-0.06	-0.01	emotion:stimsize	1.22	6	.976	0.00
emotion_happy-g.m:stimsize_medium-g.m	0.11	0.15	0.75	-0.17	0.40	0.08	0.13					
emotion_angry-g.m:stimsize_medium-g.m	-0.09	0.15	-0.59	-0.40	0.18	-0.11	-0.05					
emotion_scrambled-g.m:stimsize_large-g.m	-0.07	0.15	-0.45	-0.36	0.21	-0.09	-0.04					
emotion_happy-g.m:stimsize_large-g.m	-0.05	0.15	-0.35	-0.34	0.25	-0.08	-0.01					
emotion_angry-g.m:stimsize_large-g.m	0.10	0.15	0.69	-0.20	0.39	0.07	0.14					

Notes: β = model estimate, *SE* = standard error of the estimate, *CI* = lower and upper 95% bootstrapped confidence intervals, Stab = estimate ranges leaving out one participant at a time, LRT = Likelihood ratio test, f^2 = Cohen's f^2 effect size

Exploratory statistical models (including expression manipulation)

Table S27:
Statistical results for the P1 mean amplitudes by emotion, stimulus size and expression manipulation

	β	SE	t	CI_l	CI_u	$Stab_{min}$	$Stab_{max}$	LRT:Model	χ^2	df	p	f^2
(Intercept)	3.05	0.30	10.08	2.47	3.68	2.94	3.14	(Intercept)	-	-	-	-
emotion__happy-g.m	-0.09	0.08	-1.12	-0.22	0.06	-0.11	-0.06	emotion	2.64	2	.268	0.00
emotion__angry-g.m	0.12	0.08	1.56	-0.03	0.27	0.10	0.14					
stimsize__medium-g.m	0.11	0.08	1.38	-0.04	0.26	0.05	0.15	stimsize	5.86	2	.053	0.01
stimsize__large-g.m	0.08	0.08	1.00	-0.07	0.22	0.04	0.13					
stimprop__real-g.m	0.10	0.05	1.80	0.00	0.20	0.06	0.12	stimprop	3.31	1	.069	0.00
emotion__happy-g.m:stimsize__medium-g.m	-0.18	0.11	-1.62	-0.40	0.03	-0.21	-0.15	emotion:stimsize	3.75	4	.441	0.01
emotion__angry-g.m:stimsize__medium-g.m	0.18	0.11	1.64	-0.02	0.39	0.13	0.21					
emotion__happy-g.m:stimsize__large-g.m	0.12	0.11	1.11	-0.10	0.31	0.08	0.15					
emotion__angry-g.m:stimsize__large-g.m	-0.11	0.11	-1.02	-0.32	0.09	-0.13	-0.06					
emotion__happy-g.m:stimprop__real-g.m	0.01	0.08	0.18	-0.14	0.16	-0.01	0.04	emotion:stimprop	0.04	2	.978	0.00
emotion__angry-g.m:stimprop__real-g.m	-0.01	0.08	-0.18	-0.16	0.14	-0.03	0.00					
stimsize__medium-g.m:stimprop__real-g.m	-0.03	0.08	-0.38	-0.18	0.13	-0.05	0.00	stimsize:stimprop	0.20	2	.906	0.00
stimsize__large-g.m:stimprop__real-g.m	0.00	0.08	0.01	-0.15	0.15	-0.04	0.03					
emotion__happy-g.m:stimsize__medium-g.m:stimprop__real-g.m	0.23	0.11	2.09	0.01	0.42	0.20	0.26	emotion:stimsize:stimprop	7.93	4	.094	0.01
emotion__angry-g.m:stimsize__medium-g.m:stimprop__real-g.m	-0.17	0.11	-1.55	-0.38	0.05	-0.21	-0.13					
emotion__happy-g.m:stimsize__large-g.m:stimprop__real-g.m	0.05	0.11	0.42	-0.16	0.27	0.01	0.07					
emotion__angry-g.m:stimsize__large-g.m:stimprop__real-g.m	0.03	0.11	0.28	-0.18	0.24	0.00	0.06					

Notes: β = model estimate, SE = standard error of the estimate, CI = lower and upper 95% bootstrapped confidence intervals, Stab = estimate ranges leaving out one participant at a time, LRT = Likelihood ratio test, f^2 = Cohen's f^2 effect size

Table S28:
Statistical results for the P1 peak amplitudes by emotion, stimulus size and expression manipulation

	β	SE	t	CI_l	CI_u	$Stab_{min}$	$Stab_{max}$	LRT:Model	χ^2	df	p	f^2
(Intercept)	5.16	0.34	15.26	4.51	5.80	5.04	5.28	(Intercept)	-	-	-	-
emotion__happy-g.m	-0.12	0.08	-1.52	-0.28	0.04	-0.15	-0.10	emotion	2.67	2	.263	0.00
emotion__angry-g.m	0.10	0.08	1.23	-0.06	0.25	0.08	0.11					
stimsize__medium-g.m	0.14	0.08	1.79	-0.01	0.30	0.10	0.18	stimsize	38.55	2	<.001	0.06
stimsize__large-g.m	0.33	0.08	4.25	0.17	0.48	0.29	0.37					
stimprop__real-g.m	0.07	0.06	1.23	-0.04	0.18	0.04	0.08	stimprop	1.56	1	.212	0.00
emotion__happy-g.m:stimsize__medium-g.m	-0.19	0.11	-1.67	-0.41	0.03	-0.22	-0.15	emotion:stimsize	3.19	4	.527	0.00
emotion__angry-g.m:stimsize__medium-g.m	0.13	0.11	1.21	-0.07	0.34	0.07	0.16					
emotion__happy-g.m:stimsize__large-g.m	0.13	0.11	1.13	-0.10	0.34	0.08	0.16					
emotion__angry-g.m:stimsize__large-g.m	-0.06	0.11	-0.59	-0.28	0.15	-0.09	-0.04					
emotion__happy-g.m:stimprop__real-g.m	0.02	0.08	0.29	-0.13	0.17	0.00	0.05	emotion:stimprop	0.10	2	.953	0.00
emotion__angry-g.m:stimprop__real-g.m	-0.02	0.08	-0.22	-0.17	0.13	-0.05	0.00					
stimsize__medium-g.m:stimprop__real-g.m	-0.04	0.08	-0.55	-0.19	0.10	-0.06	0.01	stimsize:stimprop	0.67	2	.717	0.00
stimsize__large-g.m:stimprop__real-g.m	-0.02	0.08	-0.24	-0.17	0.14	-0.05	0.01					
emotion__happy-g.m:stimsize__medium-g.m:stimprop__real-g.m	0.26	0.11	2.32	0.05	0.47	0.22	0.29	emotion:stimsize:stimprop	10.32	4	.035	0.02
emotion__angry-g.m:stimsize__medium-g.m:stimprop__real-g.m	-0.17	0.11	-1.51	-0.37	0.06	-0.20	-0.11					
emotion__happy-g.m:stimsize__large-g.m:stimprop__real-g.m	0.07	0.11	0.59	-0.15	0.27	0.01	0.10					
emotion__angry-g.m:stimsize__large-g.m:stimprop__real-g.m	0.04	0.11	0.38	-0.19	0.24	0.00	0.07					

Notes: β = model estimate, SE = standard error of the estimate, CI = lower and upper 95% bootstrapped confidence intervals, Stab = estimate ranges leaving out one participant at a time, LRT = Likelihood ratio test, f^2 = Cohen's f^2 effect size

Table S29:

Statistical results for the P1 peak latency by emotion, stimulus size and expression manipulation

	β	SE	t	CI_l	CI_u	$Stab_{min}$	$Stab_{max}$	LRT:Model	χ^2	df	p	f^2
(Intercept)	99.87	1.47	68.06	96.89	102.72	99.43	100.28	(Intercept)	-	-	-	-
emotion_happy-g.m	-1.28	0.39	-3.30	-1.96	-0.50	-1.45	-1.13	emotion	11.59	2	.003	0.02
emotion_angry-g.m	0.89	0.39	2.29	0.12	1.67	0.73	1.11					
stimsize_medium-g.m	-0.57	0.39	-1.48	-1.28	0.18	-0.72	-0.40	stimsize	52.43	2	<.001	0.08
stimsize_large-g.m	-2.10	0.39	-5.42	-2.80	-1.34	-2.28	-1.81					
stimprop_real-g.m	0.41	0.27	1.51	-0.12	0.94	0.32	0.53	stimprop	2.34	1	.126	0.00
emotion_happy-g.m:stimsize_medium-g.m	0.89	0.55	1.62	-0.23	1.95	0.69	1.01	emotion:stimsize	3.13	4	.536	0.00
emotion_angry-g.m:stimsize_medium-g.m	-0.35	0.55	-0.65	-1.41	0.64	-0.52	-0.16					
emotion_happy-g.m:stimsize_large-g.m	-0.67	0.55	-1.21	-1.85	0.40	-0.79	-0.45					
emotion_angry-g.m:stimsize_large-g.m	0.47	0.55	0.86	-0.61	1.61	0.20	0.63					
emotion_happy-g.m:stimprop_real-g.m	0.18	0.39	0.47	-0.54	0.96	-0.05	0.33	emotion:stimprop	5.08	2	.079	0.01
emotion_angry-g.m:stimprop_real-g.m	-0.82	0.39	-2.12	-1.55	-0.09	-0.95	-0.71					
stimsize_medium-g.m:stimprop_real-g.m	0.46	0.39	1.19	-0.25	1.16	0.35	0.61	stimsize:stimprop	1.59	2	.452	0.00
stimsize_large-g.m:stimprop_real-g.m	-0.35	0.39	-0.91	-1.12	0.38	-0.55	-0.20					
emotion_happy-g.m:stimsize_medium-g.m:stimprop_real-g.m	-0.55	0.55	-0.99	-1.71	0.52	-0.70	-0.37	emotion:stimsize:stimprop	1.65	4	.800	0.00
emotion_angry-g.m:stimsize_medium-g.m:stimprop_real-g.m	0.46	0.55	0.84	-0.55	1.58	0.20	0.61					
emotion_happy-g.m:stimsize_large-g.m:stimprop_real-g.m	0.58	0.55	1.05	-0.48	1.59	0.38	0.73					
emotion_angry-g.m:stimsize_large-g.m:stimprop_real-g.m	-0.49	0.55	-0.89	-1.59	0.51	-0.64	-0.31					

Notes: β = model estimate, SE = standard error of the estimate, CI = lower and upper 95% bootstrapped confidence intervals, Stab = estimate ranges leaving out one participant at a time, LRT = Likelihood ratio test, f^2 = Cohen's f^2 effect sizeNote that the inspection of residuals indicated a potential misfit of the model, possibly due to the temporal boundary of the ROI time window.

Table S30:

Statistical results for the N170 mean amplitudes by emotion, stimulus size and expression manipulation

	β	SE	t	CI_l	CI_u	$Stab_{min}$	$Stab_{max}$	LRT:Model	χ^2	df	p	f^2
(Intercept)	-6.68	0.65	-10.30	-7.91	-5.44	-6.83	-6.42	(Intercept)	-	-	-	-
emotion_happy-g.m	-0.03	0.09	-0.29	-0.20	0.14	-0.05	0.00	emotion	12.87	2	.002	0.02
emotion_angry-g.m	-0.25	0.09	-2.92	-0.43	-0.08	-0.28	-0.22					
stimsize_medium-g.m	0.08	0.09	0.87	-0.11	0.26	0.02	0.10	stimsize	44.38	2	<.001	0.07
stimsize_large-g.m	0.46	0.09	5.29	0.29	0.63	0.42	0.52					
stimprop_real-g.m	-0.06	0.06	-0.98	-0.18	0.05	-0.08	-0.05	stimprop	0.98	1	.323	0.00
emotion_happy-g.m:stimsize_medium-g.m	-0.07	0.12	-0.53	-0.30	0.18	-0.12	-0.03	emotion:stimsize	0.71	4	.950	0.00
emotion_angry-g.m:stimsize_medium-g.m	-0.03	0.12	-0.21	-0.28	0.23	-0.07	0.02					
emotion_happy-g.m:stimsize_large-g.m	0.03	0.12	0.27	-0.20	0.27	-0.01	0.08					
emotion_angry-g.m:stimsize_large-g.m	-0.02	0.12	-0.14	-0.24	0.23	-0.05	0.03					
emotion_happy-g.m:stimprop_real-g.m	0.06	0.09	0.68	-0.12	0.23	0.03	0.08	emotion:stimprop	2.22	2	.330	0.00
emotion_angry-g.m:stimprop_real-g.m	-0.13	0.09	-1.47	-0.30	0.04	-0.15	-0.10					
stimsize_medium-g.m:stimprop_real-g.m	-0.12	0.09	-1.33	-0.29	0.05	-0.14	-0.07	stimsize:stimprop	2.72	2	.257	0.00
stimsize_large-g.m:stimprop_real-g.m	-0.01	0.09	-0.14	-0.19	0.15	-0.04	0.00					
emotion_happy-g.m:stimsize_medium-g.m:stimprop_real-g.m	0.19	0.12	1.50	-0.06	0.41	0.16	0.21	emotion:stimsize:stimprop	2.56	4	.634	0.00
emotion_angry-g.m:stimsize_medium-g.m:stimprop_real-g.m	-0.04	0.12	-0.35	-0.28	0.21	-0.08	-0.02					
emotion_happy-g.m:stimsize_large-g.m:stimprop_real-g.m	-0.08	0.12	-0.64	-0.32	0.16	-0.10	-0.05					
emotion_angry-g.m:stimsize_large-g.m:stimprop_real-g.m	0.02	0.12	0.16	-0.21	0.26	-0.01	0.06					

Notes: β = model estimate, SE = standard error of the estimate, CI = lower and upper 95% bootstrapped confidence intervals, Stab = estimate ranges leaving out one participant at a time, LRT = Likelihood ratio test, f^2 = Cohen's f^2 effect size

Table S31:

Statistical results for the N170 peak amplitudes by emotion, stimulus size and expression manipulation

	β	SE	t	CI_l	CI_u	$Stab_{min}$	$Stab_{max}$	LRT:Model	χ^2	df	p	f^2
(Intercept)	-11.53	0.79	-14.61	-13.15	-10.04	-11.69	-11.18	(Intercept)	-	-	-	-
emotion_happy-g.m	-0.02	0.09	-0.18	-0.19	0.17	-0.04	0.02	emotion	9.22	2	.010	0.01
emotion_angry-g.m	-0.22	0.09	-2.50	-0.40	-0.05	-0.25	-0.19					
stimsize_medium-g.m	-0.21	0.09	-2.35	-0.38	-0.04	-0.24	-0.17	stimsize	5.68	2	.058	0.01
stimsize_large-g.m	0.12	0.09	1.33	-0.05	0.29	0.08	0.17					
stimprop_real-g.m	-0.07	0.06	-1.05	-0.18	0.05	-0.10	-0.04	stimprop	1.13	1	.289	0.00
emotion_happy-g.m:stimsize_medium-g.m	0.02	0.13	0.18	-0.21	0.28	-0.03	0.05	emotion:stimsize	0.52	4	.971	0.00
emotion_angry-g.m:stimsize_medium-g.m	-0.07	0.13	-0.56	-0.33	0.17	-0.13	-0.02					
emotion_happy-g.m:stimsize_large-g.m	-0.06	0.13	-0.45	-0.30	0.17	-0.09	-0.03					
emotion_angry-g.m:stimsize_large-g.m	0.05	0.13	0.40	-0.20	0.30	0.02	0.11					
emotion_happy-g.m:stimprop_real-g.m	-0.02	0.09	-0.23	-0.20	0.16	-0.06	0.01	emotion:stimprop	4.10	2	.129	0.01
emotion_angry-g.m:stimprop_real-g.m	-0.14	0.09	-1.60	-0.32	0.04	-0.17	-0.11					
stimsize_medium-g.m:stimprop_real-g.m	-0.07	0.09	-0.77	-0.24	0.10	-0.09	-0.04	stimsize:stimprop	1.40	2	.497	0.00
stimsize_large-g.m:stimprop_real-g.m	-0.03	0.09	-0.37	-0.20	0.13	-0.06	-0.01					
emotion_happy-g.m:stimsize_medium-g.m:stimprop_real-g.m	0.13	0.13	1.02	-0.12	0.38	0.10	0.17	emotion:stimsize:stimprop	1.99	4	.738	0.00
emotion_angry-g.m:stimsize_medium-g.m:stimprop_real-g.m	-0.08	0.13	-0.65	-0.32	0.16	-0.12	-0.05					
emotion_happy-g.m:stimsize_large-g.m:stimprop_real-g.m	-0.10	0.13	-0.82	-0.34	0.14	-0.13	-0.05					
emotion_angry-g.m:stimsize_large-g.m:stimprop_real-g.m	0.14	0.13	1.13	-0.10	0.37	0.10	0.17					

Notes: β = model estimate, SE = standard error of the estimate, CI = lower and upper 95% bootstrapped confidence intervals, Stab = estimate ranges leaving out one participant at a time, LRT = Likelihood ratio test, f^2 = Cohen's f^2 effect size

Table S32:

Statistical results for the N170 peak latency by emotion, stimulus size and expression manipulation

	β	SE	t	CI_l	CI_u	$Stab_{min}$	$Stab_{max}$	LRT:Model	χ^2	df	p	f^2
(Intercept)	152.25	1.77	86.03	148.92	155.69	151.57	152.67	(Intercept)	-	-	-	-
emotion_happy-g.m	0.50	0.26	1.93	-0.07	1.01	0.41	0.58	emotion	8.80	2	.012	0.01
emotion_angry-g.m	0.24	0.26	0.94	-0.26	0.75	0.15	0.32					
stimsize_medium-g.m	-1.68	0.26	-6.52	-2.18	-1.21	-1.81	-1.61	stimsize	423.04	2	<.001	0.87
stimsize_large-g.m	-4.29	0.26	-16.64	-4.81	-3.78	-4.43	-4.13					
stimprop_real-g.m	0.33	0.18	1.81	-0.03	0.68	0.25	0.39	stimprop	3.34	1	.068	0.00
emotion_happy-g.m:stimsize_medium-g.m	-0.14	0.37	-0.37	-0.78	0.63	-0.28	-0.01	emotion:stimsize	3.44	4	.488	0.01
emotion_angry-g.m:stimsize_medium-g.m	0.28	0.36	0.78	-0.44	1.01	0.13	0.46					
emotion_happy-g.m:stimsize_large-g.m	-0.33	0.37	-0.90	-1.07	0.42	-0.44	-0.20					
emotion_angry-g.m:stimsize_large-g.m	-0.24	0.36	-0.65	-0.88	0.48	-0.34	-0.05					
emotion_happy-g.m:stimprop_real-g.m	-0.41	0.26	-1.58	-0.96	0.09	-0.59	-0.31	emotion:stimprop	2.65	2	.265	0.00
emotion_angry-g.m:stimprop_real-g.m	0.14	0.26	0.53	-0.36	0.64	0.06	0.33					
stimsize_medium-g.m:stimprop_real-g.m	-0.35	0.26	-1.34	-0.84	0.18	-0.41	-0.28	stimsize:stimprop	2.22	2	.329	0.00
stimsize_large-g.m:stimprop_real-g.m	0.31	0.26	1.20	-0.19	0.82	0.21	0.40					
emotion_happy-g.m:stimsize_medium-g.m:stimprop_real-g.m	-0.06	0.37	-0.18	-0.79	0.65	-0.18	0.05	emotion:stimsize:stimprop	1.97	4	.741	0.00
emotion_angry-g.m:stimsize_medium-g.m:stimprop_real-g.m	0.05	0.36	0.14	-0.67	0.77	-0.11	0.19					
emotion_happy-g.m:stimsize_large-g.m:stimprop_real-g.m	0.40	0.37	1.09	-0.35	1.13	0.29	0.53					
emotion_angry-g.m:stimsize_large-g.m:stimprop_real-g.m	-0.01	0.36	-0.03	-0.69	0.71	-0.16	0.07					

Notes: β = model estimate, SE = standard error of the estimate, CI = lower and upper 95% bootstrapped confidence intervals, Stab = estimate ranges leaving out one participant at a time, LRT = Likelihood ratio test, f^2 = Cohen's f^2 effect size

Table S33:

Statistical results for the EPN mean amplitudes by emotion, stimulus size and expression manipulation

	β	SE	t	CI_l	CI_u	$Stab_{min}$	$Stab_{max}$	LRT:Model	χ^2	df	p	f^2
(Intercept)	-1.20	0.58	-2.06	-2.33	-0.07	-1.49	-1.02	(Intercept)	-	-	-	-
emotion__happy-g.m	-0.30	0.09	-3.52	-0.49	-0.13	-0.33	-0.28	emotion	19.61	2	<.001	0.03
emotion__angry-g.m	-0.04	0.09	-0.51	-0.21	0.13	-0.07	-0.01					
stimsize__medium-g.m	-0.04	0.09	-0.42	-0.21	0.12	-0.06	0.00	stimsize	54.89	2	<.001	0.08
stimsize__large-g.m	0.57	0.09	6.65	0.40	0.75	0.51	0.62					
stimprop__real-g.m	-0.04	0.06	-0.63	-0.16	0.08	-0.06	-0.01	stimprop	0.41	1	.520	0.00
emotion__happy-g.m:stimsize__medium-g.m	-0.08	0.12	-0.62	-0.32	0.17	-0.11	-0.03	emotion:stimsize	3.87	4	.424	0.01
emotion__angry-g.m:stimsize__medium-g.m	0.17	0.12	1.43	-0.05	0.41	0.13	0.21					
emotion__happy-g.m:stimsize__large-g.m	0.15	0.12	1.23	-0.08	0.39	0.12	0.19					
emotion__angry-g.m:stimsize__large-g.m	-0.07	0.12	-0.59	-0.31	0.16	-0.10	-0.02					
emotion__happy-g.m:stimprop__real-g.m	-0.01	0.09	-0.14	-0.18	0.15	-0.04	0.02	emotion:stimprop	1.41	2	.494	0.00
emotion__angry-g.m:stimprop__real-g.m	-0.08	0.09	-0.94	-0.24	0.09	-0.11	-0.05					
stimsize__medium-g.m:stimprop__real-g.m	0.05	0.09	0.53	-0.12	0.21	0.02	0.07	stimsize:stimprop	0.35	2	.838	0.00
stimsize__large-g.m:stimprop__real-g.m	-0.04	0.09	-0.48	-0.21	0.13	-0.07	-0.02					
emotion__happy-g.m:stimsize__medium-g.m:stimprop__real-g.m	0.35	0.12	2.84	0.10	0.58	0.31	0.38	emotion:stimsize:stimprop	9.28	4	.055	0.01
emotion__angry-g.m:stimsize__medium-g.m:stimprop__real-g.m	-0.20	0.12	-1.68	-0.43	0.04	-0.24	-0.14					
emotion__happy-g.m:stimsize__large-g.m:stimprop__real-g.m	-0.25	0.12	-2.05	-0.47	-0.01	-0.28	-0.21					
emotion__angry-g.m:stimsize__large-g.m:stimprop__real-g.m	0.08	0.12	0.67	-0.15	0.32	0.04	0.12					

Notes: β = model estimate, *SE* = standard error of the estimate, *CI* = lower and upper 95% bootstrapped confidence intervals, Stab = estimate ranges leaving out one participant at a time, LRT = Likelihood ratio test, f^2 = Cohen's f^2 effect size

Table S34:

Statistical results for the LPC mean amplitudes by emotion, stimulus size and expression manipulation

	β	SE	t	CI_l	CI_u	$Stab_{min}$	$Stab_{max}$	LRT:Model	χ^2	df	p	f^2
(Intercept)	5.59	0.43	13.09	4.76	6.42	5.45	5.71	(Intercept)	-	-	-	-
emotion__happy-g.m	0.16	0.08	2.07	0.00	0.31	0.14	0.19	emotion	7.42	2	.024	0.01
emotion__angry-g.m	0.03	0.08	0.45	-0.11	0.18	0.00	0.07					
stimsize__medium-g.m	0.03	0.08	0.34	-0.12	0.16	0.01	0.07	stimsize	17.61	2	<.001	0.03
stimsize__large-g.m	0.26	0.08	3.42	0.11	0.40	0.22	0.28					
stimprop__real-g.m	0.03	0.05	0.54	-0.08	0.13	0.01	0.04	stimprop	0.30	1	.585	0.00
emotion__happy-g.m:stimsize__medium-g.m	0.10	0.11	0.97	-0.11	0.31	0.07	0.13	emotion:stimsize	3.44	4	.487	0.01
emotion__angry-g.m:stimsize__medium-g.m	-0.10	0.11	-0.93	-0.29	0.12	-0.13	-0.05					
emotion__happy-g.m:stimsize__large-g.m	-0.18	0.11	-1.72	-0.40	0.02	-0.21	-0.13					
emotion__angry-g.m:stimsize__large-g.m	0.09	0.11	0.82	-0.12	0.30	0.05	0.12					
emotion__happy-g.m:stimprop__real-g.m	0.01	0.08	0.18	-0.14	0.17	-0.02	0.04	emotion:stimprop	0.03	2	.983	0.00
emotion__angry-g.m:stimprop__real-g.m	-0.01	0.08	-0.08	-0.16	0.14	-0.03	0.03					
stimsize__medium-g.m:stimprop__real-g.m	-0.09	0.08	-1.15	-0.24	0.05	-0.13	-0.06	stimsize:stimprop	1.52	2	.467	0.00
stimsize__large-g.m:stimprop__real-g.m	0.02	0.08	0.22	-0.13	0.17	-0.01	0.05					
emotion__happy-g.m:stimsize__medium-g.m:stimprop__real-g.m	-0.10	0.11	-0.89	-0.32	0.11	-0.13	-0.06	emotion:stimsize:stimprop	2.12	4	.714	0.00
emotion__angry-g.m:stimsize__medium-g.m:stimprop__real-g.m	0.10	0.11	0.97	-0.10	0.32	0.06	0.13					
emotion__happy-g.m:stimsize__large-g.m:stimprop__real-g.m	0.07	0.11	0.63	-0.13	0.28	0.01	0.11					
emotion__angry-g.m:stimsize__large-g.m:stimprop__real-g.m	-0.14	0.11	-1.27	-0.34	0.07	-0.17	-0.11					

Notes: β = model estimate, *SE* = standard error of the estimate, *CI* = lower and upper 95% bootstrapped confidence intervals, Stab = estimate ranges leaving out one participant at a time, LRT = Likelihood ratio test, f^2 = Cohen's f^2 effect size

Table S35:

Statistical results for mean response times by emotion, stimulus size and expression manipulation

	β	SE	t	CI_l	CI_u	$Stab_{min}$	$Stab_{max}$	LRT:Model	χ^2	df	p	f^2
(Intercept)	1041.52	44.14	23.60	951.17	1120.29	1023.36	1054.24	(Intercept)	-	-	-	-
emotion_happy-g.m	-36.51	5.40	-6.76	-47.57	-25.56	-38.60	-33.78	emotion	80.70	2	<.001	0.13
emotion_angry-g.m	47.06	5.40	8.71	36.58	57.14	44.37	48.63					
stimsize_large-g.m	13.32	5.40	2.47	2.85	23.85	11.15	15.11	stimsize	6.43	2	.040	0.01
stimsize_medium-g.m	-8.84	5.40	-1.64	-18.72	1.71	-11.19	-5.92					
stimprop_real-g.m	3.10	3.82	0.81	-4.30	10.58	1.87	6.68	stimprop	0.67	1	.411	0.00
emotion_happy-g.m:stimsize_large-g.m	-7.11	7.64	-0.93	-22.57	7.02	-9.79	-4.83	emotion:stimsize	6.87	4	.143	0.01
emotion_angry-g.m:stimsize_large-g.m	-2.07	7.64	-0.27	-16.06	13.54	-4.47	2.14					
emotion_happy-g.m:stimsize_medium-g.m	17.15	7.64	2.24	2.06	32.33	13.71	18.76					
emotion_angry-g.m:stimsize_medium-g.m	-11.27	7.64	-1.47	-26.94	3.08	-13.26	-7.65					
emotion_happy-g.m:stimprop_real-g.m	-0.73	5.40	-0.13	-11.23	9.62	-5.15	2.95	emotion:stimprop	3.50	2	.174	0.01
emotion_angry-g.m:stimprop_real-g.m	-8.27	5.40	-1.53	-18.96	2.58	-10.83	-5.57					
stimsize_large-g.m:stimprop_real-g.m	1.67	5.40	0.31	-8.99	12.05	-0.73	3.77	stimsize:stimprop	2.22	2	.330	0.00
stimsize_medium-g.m:stimprop_real-g.m	5.90	5.40	1.09	-5.43	16.10	3.50	8.20					
emotion_happy-g.m:stimsize_large-g.m:stimprop_real-g.m	1.27	7.64	0.17	-13.45	14.97	-2.06	3.62	emotion:stimsize:stimprop	1.94	4	.746	0.00
emotion_angry-g.m:stimsize_large-g.m:stimprop_real-g.m	-4.59	7.64	-0.60	-19.58	10.28	-7.73	0.02					
emotion_happy-g.m:stimsize_medium-g.m:stimprop_real-g.m	2.24	7.64	0.29	-11.93	16.92	0.05	5.42					
emotion_angry-g.m:stimsize_medium-g.m:stimprop_real-g.m	7.46	7.64	0.98	-7.37	22.14	4.26	9.93					

Notes: β = model estimate, *SE* = standard error of the estimate, *CI* = lower and upper 95% bootstrapped confidence intervals, Stab = estimate ranges leaving out one participant at a time, LRT = Likelihood ratio test, f^2 = Cohen's f^2 effect size

Table S36:

Statistical results for the probability of correct responses in the naturalness classification task by emotion, stimulus size and expression manipulation

	β	SE	t	CI_l	CI_u	$Stab_{min}$	$Stab_{max}$	LRT:Model	χ^2	df	p	f^2
(Intercept)	0.14	0.06	2.35	0.02	0.26	0.12	0.19	(Intercept)	-	-	-	-
emotion_happy-g.m	0.34	0.06	6.12	0.23	0.45	0.52	0.60	emotion	28.21	2	<.001	0.07
emotion_angry-g.m	0.00	0.03	-0.10	-0.07	0.06	-0.10	-0.04					
stimsize_large-g.m	0.01	0.03	0.18	-0.05	0.06	-0.13	-0.06	stimsize	0.10	2	.952	0.00
stimsize_medium-g.m	0.01	0.03	0.16	-0.05	0.07	-0.13	-0.05					
stimprop_real-g.m	0.20	0.08	2.64	0.05	0.36	0.39	0.49	stimprop	6.42	1	.011	0.04
emotion_happy-g.m:stimsize_large-g.m	0.05	0.03	1.96	0.00	0.11	0.06	0.19	emotion:stimsize	15.94	4	.003	0.00
emotion_angry-g.m:stimsize_large-g.m	0.05	0.03	1.79	0.00	0.10	0.03	0.15					
emotion_happy-g.m:stimsize_medium-g.m	-0.02	0.03	-0.59	-0.07	0.04	-0.02	0.13					
emotion_angry-g.m:stimsize_medium-g.m	0.00	0.03	-0.14	-0.06	0.05	-0.15	-0.03					
emotion_happy-g.m:stimprop_real-g.m	0.19	0.02	9.92	0.15	0.23	0.26	0.41	emotion:stimprop	234.52	2	<.001	0.04
emotion_angry-g.m:stimprop_real-g.m	-0.29	0.02	-15.03	-0.32	-0.25	-0.54	-0.37					
stimsize_large-g.m:stimprop_real-g.m	-0.10	0.02	-5.36	-0.14	-0.07	-0.18	-0.10	stimsize:stimprop	55.27	2	<.001	0.01
stimsize_medium-g.m:stimprop_real-g.m	-0.03	0.02	-1.77	-0.07	0.00	-0.08	0.01					
emotion_happy-g.m:stimsize_large-g.m:stimprop_real-g.m	0.07	0.03	2.36	0.01	0.12	0.18	0.30	emotion:stimsize:stimprop	24.69	4	<.001	0.00
emotion_angry-g.m:stimsize_large-g.m:stimprop_real-g.m	-0.02	0.03	-0.61	-0.07	0.03	-0.08	0.01					
emotion_happy-g.m:stimsize_medium-g.m:stimprop_real-g.m	0.06	0.03	2.25	0.01	0.12	0.11	0.22					
emotion_angry-g.m:stimsize_medium-g.m:stimprop_real-g.m	0.00	0.03	-0.10	-0.06	0.05	-0.21	-0.11					

Notes: beta = model estimate, SE = standard error of the estimate, CI = lower and upper 95 Stab = estimate ranges leaving out one participant at a time, LRT = Likelihood ratio test

Signal detection theory: Analysis of real-fake decisions

GLMER Model (response model) to estimate d' and c

- For similar approaches, see Wright and London (2009). For a more general introduction, see Hautus, Macmillan, and Creelman (2021), specifically p. 322 for generalized linear models in the SDT context.
- The model uses sum contrast coding (also known as deviation coding). Factor levels of the variables emotion are "angry", "happy" and "neutral"(reference), and of stimulus size "large", "medium", and "small"(reference).
- Formula: glmer(saysreal ~ isrealcontr * emotion * stimsize + (1+ isrealcontr + emotion + stimsize|sub_id), family = binomial(link ="probit"), data = df_sdt)
- Please note that the variable `isrealcontr` refers to the variable that indicates whether a real or artificial face was presented [1]. This variable was re-coded to indicate -.5 for fake faces and .5 for real faces. This makes the intercept multiplied by -1 correspond to the average criterion and the estimate β of `isrealcontr` correspond to the average d'.
- Compare with the obtained values for averaged d' = 0.16 and averaged criterion c = -0.12 calculated with the standard formulas:
  - dprime $d' = z(H) - z(F)$, with hitrate H,false alarm rate F, and the function $z(\cdot)$ being the inverse of the normal cumulative distribution function.
  - criterion $c = -\frac{1}{2}(z(H) + z(F))$

Table S37:
Statistical results for the SDT generalized linear probit model

	β	SE	z	p	LRT:Model	χ^2	df	p
(Intercept)	0.13	0.05	2.69	0.01	(Intercept)	-	-	-
isrealcontr	0.16	0.08	2.03	0.04	isrealcontr	0.00	0	-
emotion1	0.06	0.06	0.96	0.33	emotion	5.40	2	.067
emotion2	-0.19	0.08	-2.35	0.02				
stimsize1	-0.07	0.02	-3.08	0.00	stimsize	9.46	2	.009
stimsize2	-0.02	0.03	-0.95	0.34				
isrealcontr:emotion1	-0.41	0.02	-16.76	0.00	isrealcontr:emotion	0.00	0	-
isrealcontr:emotion2	-0.01	0.02	-0.28	0.78				
isrealcontr:stimsize1	0.01	0.02	0.39	0.70	isrealcontr:stimsize	0.00	0	-
isrealcontr:stimsize2	0.02	0.02	0.75	0.45				
emotion1:stimsize1	-0.04	0.02	-2.40	0.02	emotion:stimsize	29.03	4	<.001
emotion2:stimsize1	0.00	0.02	-0.01	0.99				
emotion1:stimsize2	-0.04	0.02	-2.27	0.02				
emotion2:stimsize2	0.00	0.02	-0.11	0.92				
isrealcontr:emotion1:stimsize1	-0.13	0.03	-3.71	0.00	isrealcontr:emotion:stimsize	17.29	4	.002
isrealcontr:emotion2:stimsize1	0.06	0.03	1.61	0.11				
isrealcontr:emotion1:stimsize2	0.01	0.03	0.24	0.81				
isrealcontr:emotion2:stimsize2	0.01	0.03	0.22	0.82				

Notes: β = model estimate, SE = standard error of the estimate, LRT = Likelihood ratio test

[1] this variable name was chosen to be explicit about the reference level. In the other models, we called this variable `stimprop`

Table S38:
Estimated SDT parameters discriminability index (d') and criterion (c)

stimulus size	emotion	c	SE	p	d'	SE	p
large	neutral	-0.08	0.09	.393	-0.37	0.09	<.001
	angry	0.13	0.09	.128	0.22	0.09	.018
	happy	-0.23	0.09	.010	0.66	0.09	<.001
medium	neutral	-0.13	0.10	.211	-0.22	0.09	.019
	angry	0.08	0.09	.347	0.18	0.09	.052
	happy	-0.28	0.09	.002	0.58	0.09	<.001
small	neutral	-0.37	0.09	<.001	-0.16	0.09	.098
	angry	-0.04	0.09	.630	0.06	0.09	.492
	happy	-0.28	0.09	.003	0.49	0.09	<.001

Creating fake expressions

Figure S2:

Illustration of the creation of expressive faces for the present study.

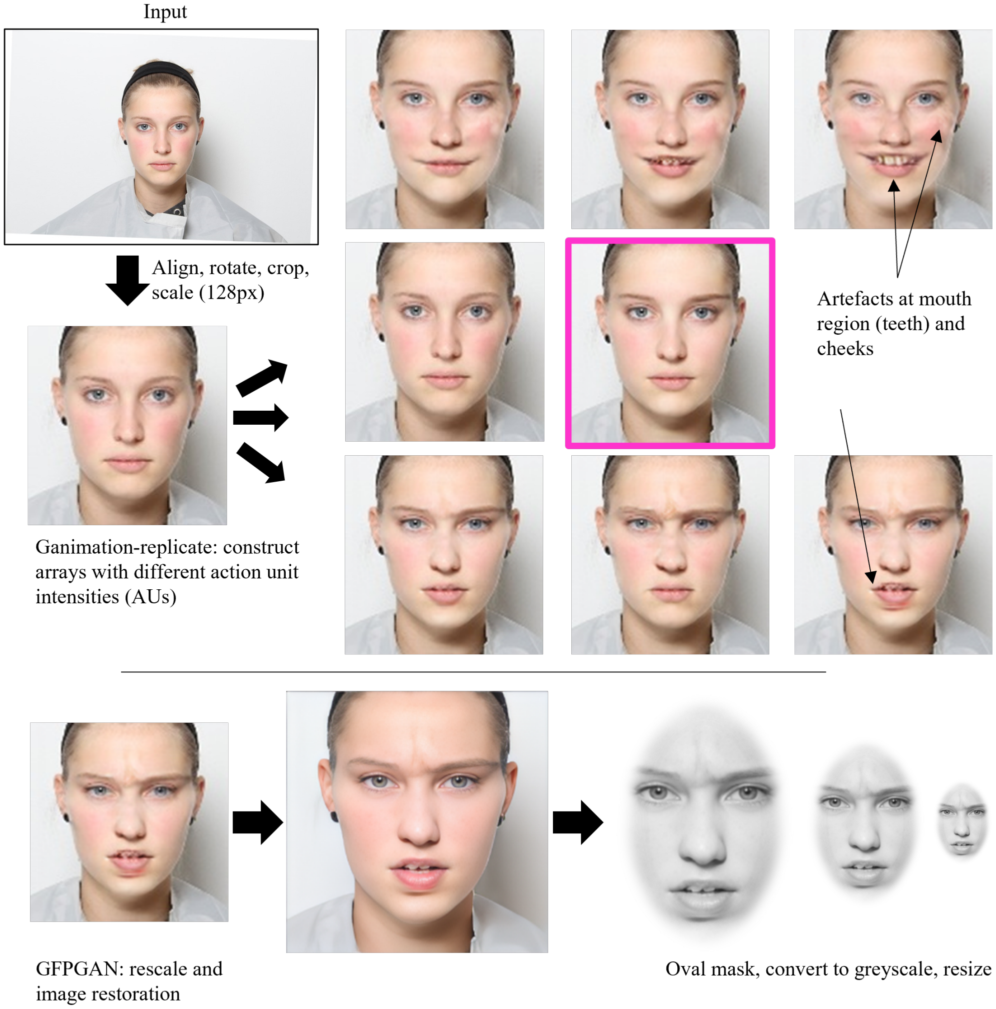

Notes: Stimulus processing starts with alignment, rotation, scaling and cropping of the input image, automatically with *OpenFace*. The 128 $\times$ 128 px image is the input for the first generative adversarial network (GAN), 'Ganimation-replicate'. An array of intensities for 17 available action units has to be specified and passed to the model. Some example outputs are displayed on the right. With increasing intensity, the model produced artefacts, especially at the mouth and cheek region for happy and angry stimuli. Some were diminshed by the next step, restoring and upscaling the expressive images using GFPGAN. Finally, an oval mask with gaussian blur was applied to the image, the image was normalized and resized. Note, that we also created instances of model-based neutral expressions with the first GAN (highlighted in pink).The photograph was taken from the Goettingen Faces Database (Kulke et al., 2017). Permission obtained.

Image properties

Figure S3:
Quantiles of pixel RGB values as a measure of luminance per stimulus category.

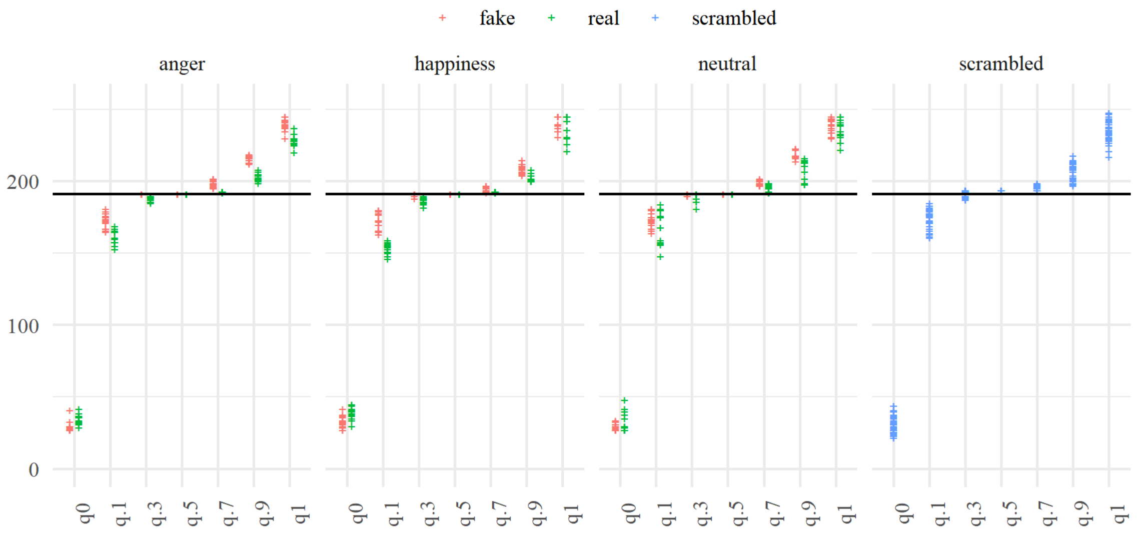

Notes: Every cross represents an individual stimulus. The black horizontal line indicates the background luminance which corresponded to the median of stimuli. Medians of scrambled versions had a slight positive offset (+3). Mean luminance and contrast differed between stimuli depicting fake and real expressions despite normalization.

Figure S4:
Averaged power spectrum of the spatial frequencies for intact and scrambled faces.

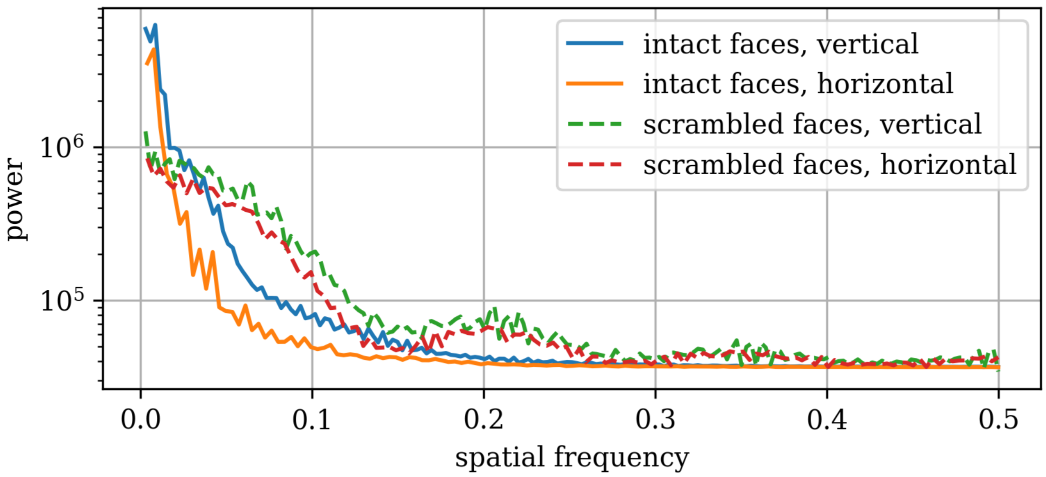

Notes: Scrambling by shuffling chunks of pixels resulted in a different power spectrum and importantly, did not preserve the pronounced low frequencies of the intact facial stimuli.

The perception of artificiality of faces (Tauscher et al. 2021; Tucciarelli et al. 2022) were not the main focus of this study. Hence, we planned to investigate and discuss them separately, as they raise a timely and intriguing research question in their own. Yet, potentially relevant factors that may have contributed to the present findings are that real and manipulated happy (more than angry and neutral) faces differed in several dimensions that participants may have learned to use as discriminative cues over the course of the experiment. These potential cues to realness included a) the overall variability of expression intensities, b) the variability of the mouth region (particularly the teeth, as the GAN was unaware of how

the person's teeth actually looked like), and the eye region, with stronger activation of the orbicularis oculi (cheek raiser) influencing the perceived intensity of the smile (Gunnery and Ruben 2015). Non-expressive cues were the illumination of the face due to the different lighting conditions between the databases, some of which remained in the processed stimuli. Learning about these cues could have triggered a focus on specific facial features, potentially biasing not only behavioral responses. For example, LPC effects may have been partly caused by different processing demands and motivation to integrate indicative facial cues, which was probably easier for happy and more difficult for neutral expressions.

References

Bar, M., K. S. Kassam, A. S. Ghuman, J. Boshyan, A. M. Schmid, A. M. Dale, M. S. Hämäläinen, et al. 2006. "Top-down Facilitation of Visual Recognition." *Proceedings of the National Academy of Sciences* 103 (2): 449–54. https://doi.org/10.1073/pnas.0507062103.

Bentin, Shlomo, Truett Allison, Aina Puce, Erik Perez, and Gregory McCarthy. 1996. "Electrophysiological Studies of Face Perception in Humans." *Journal of Cognitive Neuroscience* 8 (6): 551–65. https://doi.org/10.1162/jocn.1996.8.6.551.

Cesarei, Andrea De, and Maurizio Codispoti. 2013. "Spatial Frequencies and Emotional Perception." *Reviews in the Neurosciences* 24 (1). https://doi.org/10.1515/revneuro-2012-0053.

Gliga, Teodora, and Ghislaine Dehaene-Lambertz. 2005. "Structural Encoding of Body and Face in Human Infants and Adults." *Journal of Cognitive Neuroscience* 17 (8): 1328–40. https://doi.org/10.1162/0898929055002481.

Gunnery, Sarah D., and Mollie A. Ruben. 2015. "Perceptions of Duchenne and Non-Duchenne Smiles: A Meta-Analysis." *Cognition and Emotion* 30 (3): 501–15. https://doi.org/10.1080/02699931.2015.1018817.

Hautus, Michael J., Neil A. Macmillan, and C. Douglas Creelman. 2021. *Detection Theory: A User's Guide*. Routledge. https://doi.org/10.4324/9781003203636.

Herrmann, M. J., A.-C. Ehlis, H. Ellgring, and A. J. Fallgatter. 2004. "Early Stages (P100) of Face Perception in Humans as Measured with Event-Related Potentials (ERPs)." *Journal of Neural Transmission* 112 (8): 1073–81. https://doi.org/10.1007/s00702-004-0250-8.

Johannes, S., T. F. Münte, H. J. Heinze, and G. R. Mangun. 1995. "Luminance and Spatial Attention Effects on Early Visual Processing." *Cognitive Brain Research* 2 (3): 189–205. https://doi.org/10.1016/0926-6410(95)90008-x.

Kulke, Louisa, Laura Janßen, Ronja Demel, and Annekathrin Schacht. 2017. "Validating the Goettingen Faces Database." *OSF*. Open Science Framework. https://doi.org/10.17605/OSF.IO/4KNPF.

Mercure, Evelyne, Frederic Dick, Hanife Halit, Jordy Kaufman, and Mark H. Johnson. 2008. "Differential Lateralization for Words and Faces: Category or Psychophysics?" *Journal of Cognitive Neuroscience* 20 (11): 2070–87. https://doi.org/10.1162/jocn.2008.20137.

Ruiz-Soler, Marcos, and Francesc S. Beltran. 2005. "Face Perception: An Integrative Review of the Role of Spatial Frequencies." *Psychological Research Psychologische Forschung* 70 (4): 273–92. https://doi.org/10.1007/s00426-005-0215-z.

Schindler, Sebastian, Maximilian Bruchmann, Bettina Gathmann, Robert Moeck, and Thomas Straube. 2021. "Effects of low-level visual information and perceptual load on P1 and N170 responses to emotional expressions." *Cortex* 136 (March): 14–27. https://doi.org/10.1016/j.cortex.2020.12.011.

Schindler, Sebastian, Clara Tirloni, Maximilian Bruchmann, and Thomas Straube. 2021. "Face and Emotional Expression Processing Under Continuous Perceptual Load Tasks: An ERP Study." *Biological Psychology* 161 (April): 108056. https://doi.org/10.1016/j.biopsycho.2021.108056.

Tauscher, Jan-Philipp, Susana Castillo, Sebastian Bosse, and Marcus Magnor. 2021. "EEG-Based Analysis of the Impact of Familiarity in the Perception of Deepfake Videos." In *2021 IEEE International Conference on Image Processing (ICIP)*. IEEE. https://doi.org/10.1109/icip42928.2021.9506082.

Tucciarelli, Raffaele, Neza Vehar, Shamil Chandaria, and Manos Tsakiris. 2022. "On the Realness of People Who Do Not Exist: The Social Processing of Artificial Faces." *iScience*, December, 105441. https://doi.org/10.1016/j.isci.2022.105441.

Willenbockel, Verena, Franco Lepore, Dang Khoa Nguyen, Alain Bouthillier, and Frédéric Gosselin. 2012. "Spatial Frequency Tuning During the Conscious and Non-Conscious Perception of Emotional Facial Expressions – an Intracranial ERP Study." *Frontiers in Psychology* 3. https://doi.org/10.3389/fpsyg.2012.00237.

Wright, Daniel B., and Kamala London. 2009. "Multilevel Modelling: Beyond the Basic Applications." *British Journal of Mathematical and Statistical Psychology* 62 (2): 439–56. https://doi.org/https://doi.org/10.1348/000711008X327632.

Zion-Golumbic, E., and S. Bentin. 2006. "Dissociated Neural Mechanisms for Face Detection and Configural Encoding: Evidence from N170 and Induced Gamma-Band Oscillation Effects." *Cerebral Cortex* 17 (8): 1741–49. https://doi.org/10.1093/cercor/bhl100.
